# Discovery of a tribenzophenazine analog for binding to the KRAS mRNA G-quadruplex structures in the cisplatin-resistant non–small cell lung cancer

**DOI:** 10.1016/j.jbc.2025.108164

**Published:** 2025-01-08

**Authors:** Xiao-Dong Wang, Jia-Hong Lin, Ming-Hao Hu

**Affiliations:** Nation-Regional Engineering Lab for Synthetic Biology of Medicine, International Cancer Center, School of Pharmacy, Shenzhen University Medical School, Shenzhen, China

**Keywords:** NSCLC, KRAS, RNA G-quadruplex, tribenzophenazine

## Abstract

Lung cancer is the malignant tumor with the highest morbidity and mortality rate worldwide, of which non–small cell lung cancer (NSCLC) accounts for approximately 85%. KRAS mutations are one of the significant mechanisms underlying the occurrence, development, immune escape, and chemotherapy resistance of NSCLC. Two KRAS inhibitors are approved by Food and Drug Administration for the treatment of NSCLC in the past 3 years. However, they are only effective to KRAS G12C mutant, and moreover, innate and acquired drug resistance is already reported, leaving an urgent need to block KRAS pathways through novel targets. In this study, we focused on the discovery of ligands targeting the RNA G-quadruplexes in 5′-UTR of KRAS mRNA, and a novel tribenzophenazine analog (MBD) was identified as the lead compound. Further mechanisms were discussed in A549/DDP cells, a cisplatin-resistant and KRAS-mutant NSCLC cell line. Antitumor efficacy was verified both *in vitro* in A549/DDP cells, and *in vivo* in a nude mouse xenograft model implanted with A549/DDP cells. To sum up, our results suggest the potential of MBD as a prominent anti-KRAS–driven NSCLC agent and propose a new idea for the development of small molecule ligands targeting KRAS RNA G-quadruplexes.

Lung cancer is a type of malignant tumor with high morbidity and mortality, making the most common cause of cancer-related deaths in men and second most common in women after breast cancer. Non–small cell lung cancer (NSCLC) accounts for 85% of the lung cancer, whereas the standard care for early-stage NSCLC is surgery and chemotherapy. Over the past decade, the treatment of NSCLC develops rapidly, including target therapy, antiangiogenic therapy and immune checkpoint inhibitors (1, 2). However, only platinum-based chemotherapy and immunotherapy are applied for the patients of advanced-stage tumor without a clinically actionable alteration, and few clinically approved options exist during the tumor progression (3). Meanwhile, drug resistance emerges along with the treatment of NSCLC, becoming a huge challenge.

RAS signaling has an essential role in driving normal physiological cellular proliferation, and RAS mutations or amplifications are among the most frequent abnormality in human cancers (4). KRAS mutations are often present in solid tumors and account for 78% of all RAS mutations found in NSCLC (5). Mutations at glycine 12 of KRAS are most common, while glycine 13 is the second most frequently affected residue. KRAS codon 12 mutations lead to G12C, G12V, or G12D substitutions, accounting for 40%, 19%, and 15% of KRAS mutations in NSCLC, respectively (6). KRAS protein primarily binds to GDP in an inactive confirmation and passively loads with GTP to an active confirmation (7). The activated state of KRAS accumulating *in vivo* allows multiple downstream effector pathways, like MAPK and PI3K pathways, which are closely related to tumorigenesis, tumor progression, immune escape, and chemotherapy resistance (8).

KRAS mutations are considered as “undruggable” for more than 40 years, until Food and Drug Administration approves two KRAS inhibitors, sotorasib and adagrasib, for the treatment of advanced-stage NSCLC in the past 3 years (9). Sotorasib is a first-in-class small molecule inhibitor targeting KRAS G12C, specifically and irreversibly locking KRAS in an inactive GDP-loaded “off” state (10). Adagrasib is another small molecule KRAS inhibitor with high selectivity to mutant cysteine 12 of KRAS (11). In spite of the remarkable clinical responses of KRAS G12C inhibitors, innate and acquired drug resistance is already reported, mainly due to KRAS alterations and amplifications, as well as feedback activation of KRAS upstream and downstream signaling pathways (7, 12). Besides, KRAS oncogenic addiction widely exists in numerous cancer types, which might result in the failure of KRAS G12C inhibitors (13). More importantly, KRAS inhibitors approved for human use are only effective to KRAS G12C mutant, leaving challenging targets of other KRAS mutants for drug development. Recently, MRTX1133, a selective KRAS G12D inhibitor in clinical trial, is identified and proved to be efficacious in pancreatic ductal adenocarcinoma model through the regulation of tumor microenvironment (14, 15). BI-2865, a pan-KRAS inhibitor, is reported to prevent the activation of WT KRAS and a broad range of KRAS mutants, leading to tumor growth suppression in mice (16). Therefore, investigations of small molecule inhibitors are still of great importance, especially blocking KRAS pathways through novel targets and mechanisms.

G-quadruplexes (G4s) are a type of highly stable nucleic acid secondary structure, involving in a variety of biological functions. G4s have been identified in the telomeric and promoter regions of DNA, in charge of genome stability and gene transcription (17). Plenty of DNA G4 binders are developed to study the physical properties of G4s in cells, and their antitumor effects and mechanisms are also thoroughly determined (18, 19, 20). Recently, RNA G4s (RG4s) present in 5′-UTR of mRNA attract increasing attention, since more thermodynamically stable structures are formed for RNA G4s than DNA G4s (21, 22). The presence of G4s in 5′-UTR plays an essential role in preventing the translation of mRNA, by the inhibition of either the assembling of the translation initiation complex at 5′-cap or the scanning of the ribosome toward AUG codon (23, 24). Recent report demonstrates that 5′-UTR of KRAS mRNA contains repetitive runs of two guanines for 192 nucleotides (Fig. 1*A*), with the potential to fold into several G4s. TMPyP4, a typical G4 ligand, binds to the RG4 structures and leads to downregulation of KRAS in pancreatic cancer cells (25). Anthrafurandione and anthrathiophenedione analogs with the ability to target KRAS RG4s under low abundance cellular conditions are also proved to repress the translation in a dose-dependent manner (26). Thus, small molecule ligands targeting KRAS RG4s would be an effective therapeutic strategy for KRAS-driven NSCLC, especially tumors with chemotherapy resistance. However, few such ligands have been investigated, let alone their antitumor potential against KRAS-driven tumors. Indeed, there is an urgent need to discover KRAS RG4-targeted ligands with novel scaffolds and explore their anti-NSCLC mechanisms.

**Figure 1:**
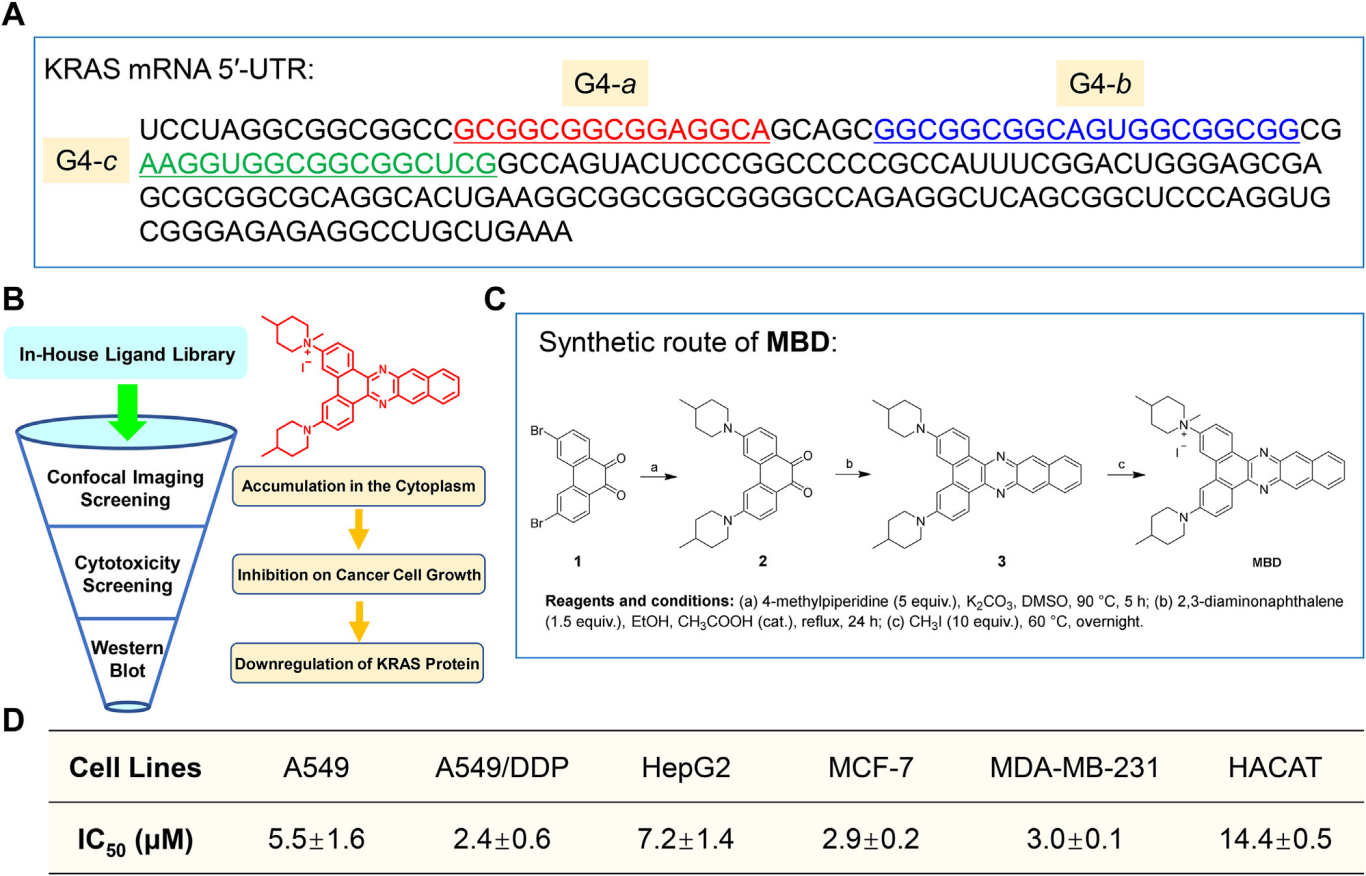
**Discovery of MBD as a KRAS mRNA repressor.***A*, the sequence of 5′-UTR of KRAS mRNA, with the potential to fold into several G4s. *B*, screening scheme for KRAS mRNA repressors in our in-house G4 ligand library. *C*, synthesis route of MBD. *D*, cytotoxicity of MBD (24 h treatment) on types of cancer cell lines as well as normal human keratinocyte line, decided by CCK8 assay. G4, G-quadruplex.

In this study, we hierarchically screened an in-house G4 ligand library designed and synthesized by our group. A novel tribenzophenazine analog (MBD) was identified as the most promising ligand toward KRAS RG4s, with the capability of accumulating in the cytoplasm, inhibiting cell growth, and decreasing KRAS expression. Interestingly, this ligand belonged to the quinoxaline scaffold developed by our group (27, 28, 29, 30, 31), which presented as a novel cation form. Therefore, antitumor efficacy of MBD was confirmed in A549/DDP cell line, which is a human NSCLC cell line carrying KRAS G12S mutation, and resistant to a typical chemotherapeutic drug (DDP, cisplatin). Interaction of MBD with KRAS RG4s was determined by CD, fluorescence and NMR spectroscopy. MBD was further proved to induce RG4s in A549/DDP cells and downregulate the expression of KRAS protein and the activation of KRAS downstream signaling pathways. Finally, MBD was demonstrated to suppress tumor growth *in vivo* in a nude mouse xenograft model implanted with A549/DDP cells. Our results displayed that MBD, a small molecule compound with a novel scaffold, was a promising therapeutic agent against cisplatin-resistant NSCLC, providing new insight into the design of binders targeting KRAS RG4s.

## Results and discussion

### Discovery of MBD as a potential KRAS mRNA repressor

Compounds targeting KRAS mRNA should possess the ability to penetrate the cell membrane and accumulate in the cell cytoplasm, and then repress the translation of mRNA to protein. We hierarchically screened an in-house G4 ligand library of about 300 compounds designed and synthesized by our group. Accumulation within the cytoplasm was initially confirmed through confocal imaging, followed by the determination of cancer cell growth inhibition using IC_50_ values below 10 μM. Ultimately, downregulation of KRAS protein expression was verified through Western blot analysis (Fig. 1*B*). MBD turned out to be the most potent G4 ligand targeting KRAS mRNA after all of the above screening. The synthesis route of MBD was displayed in Figure 1*C*, with the structure of the compound confirmed by ^1^H and ^13^C NMR spectrometry.

Next, cytotoxicity of MBD was further confirmed by Cell Counting Kit-8 (CCK8) assay, with the results displayed in Figure 1*D*. MBD inhibited the proliferation of several types of tumor cells, where IC_50_ values were commonly smaller than 10 μM. Besides, a relatively higher cytotoxicity was obtained in A549/DDP cells with an IC_50_ of 2.4 μM, which are a type of NSCLC cells with a KRAS mutation, which are resistance to DDP (cisplatin) (32). Notably, we observed that MBD displayed a relatively lower cytotoxicity on the normal human keratinocyte line (HACAT) with an IC_50_ of 14.4 μM. These findings demonstrated that MBD may serve as a potential therapeutic agent for the cisplatin-resistant NSCLC.

### Interaction of MBD with KRAS RG4 structures

**Figure 2:**
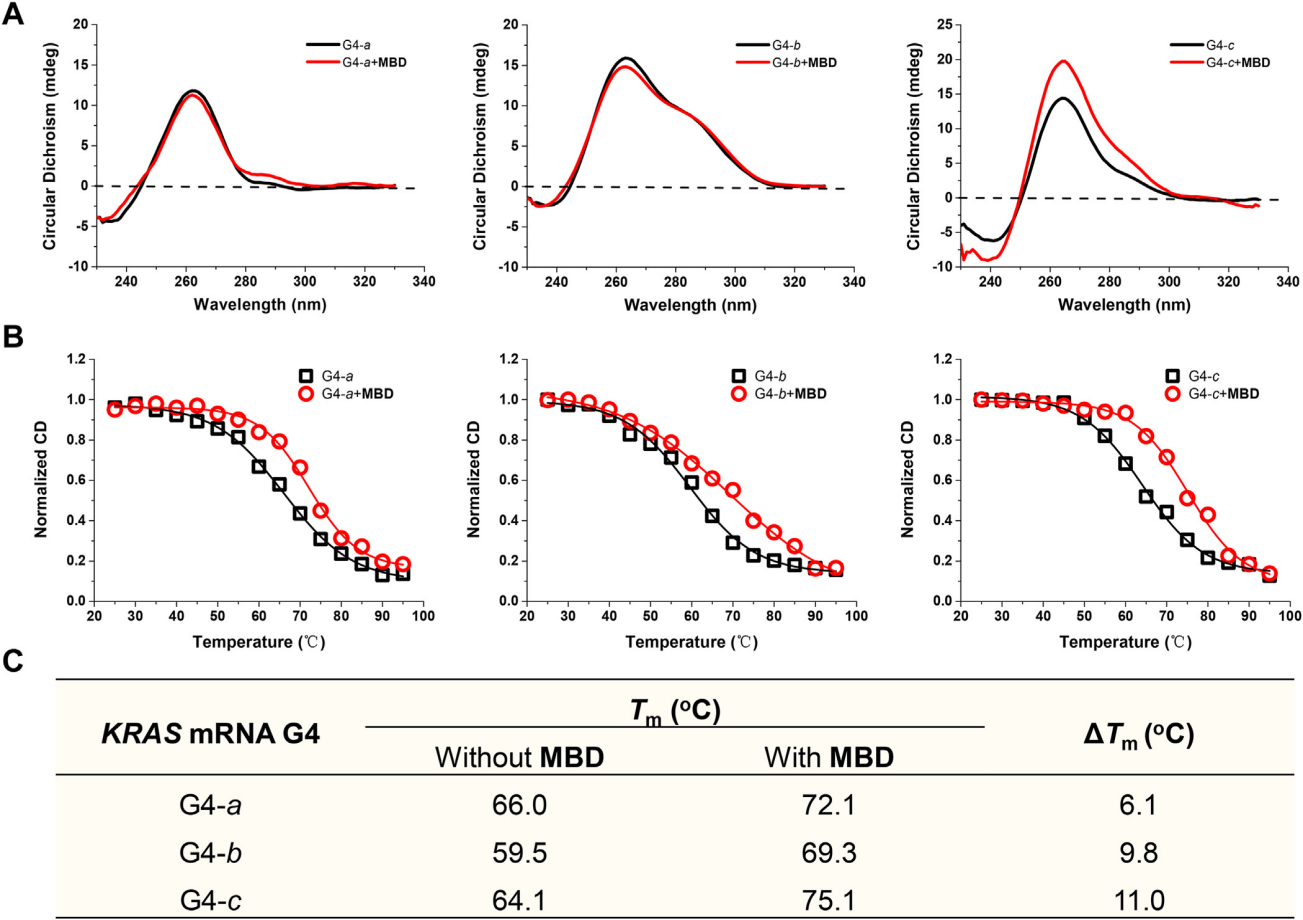
**Interaction of MBD with three types of KRAS RG4 structures.***A*, CD spectra of KRAS RNA G4-a, G4-b, and G4-c alone (*black*) and in the presence of MBD (*red*). *B*, CD melting assay of KRAS RNA G4-a, G4-b, and G4-c alone (*black*) and in the presence of MBD (*red*). *C*, *T*_m_ and Δ*T*_m_ values of G4s induced by MBD were summarized in this table. Concentrations of RNA samples and MBD were 2 μM and 10 μM, respectively. RG4, RNA G-quadruplex.

The 5′-UTR of the human KRAS transcript contains G4 motifs capable of forming RG4 structures, which can serve as targets for small ligands (25, 33). Specifically, a G-rich sequence comprising 192 nucleotides with a 77% GC content is present in this region. Within the first 80 nucleotides of the 5′-UTR, three nonoverlapping G4 motifs have been confirmed, each possessing the capacity to form G4 structures comprising two G-tetrads (26). Here, we examined the interaction of MBD to these three types of KRAS RG4 structures, that is, KRAS RNA G4-a, G4-b, and G4-c (sequences shown in Table S1), and CD assay was first employed to investigate the binding properties. As expected, all of the KRAS RG4 motifs displayed positive bands at 265 nm and negative bands at 240 nm, indicating the occurrence of typical parallel G4s. CD spectra were also recorded immediately after addition of the compound. As shown in Figure 2*A*, no obvious induced shift of KRAS RG4s could be observed, whereas only the peak intensity of KRAS RNA G4-c was increased by MBD. Thus, MBD did not alter the conformation of KRAS RG4 structures.

The stabilizing ability of the compound on KRAS RG4 structures was studied by CD melting assay, and the results were shown in Figure 2, *B* and *C*. *T*_m_ values of KRAS RNA G4-a, G4-b and G4-c alone were 66.0 °C, 59.5 °C, and 64.1 °C, respectively. MBD elevated the *T*_m_ values of all of the KRAS RG4 motifs, with the highest Δ*T*_m_ value of 11.0 °C for KRAS RNA G4-c. The above results proved the most potent stabilizing ability of MBD to KRAS RNA G4-c, so KRAS RNA G4-c was applied in the following experiments, as a representative of KRAS RG4 structures.

**Figure 3:**
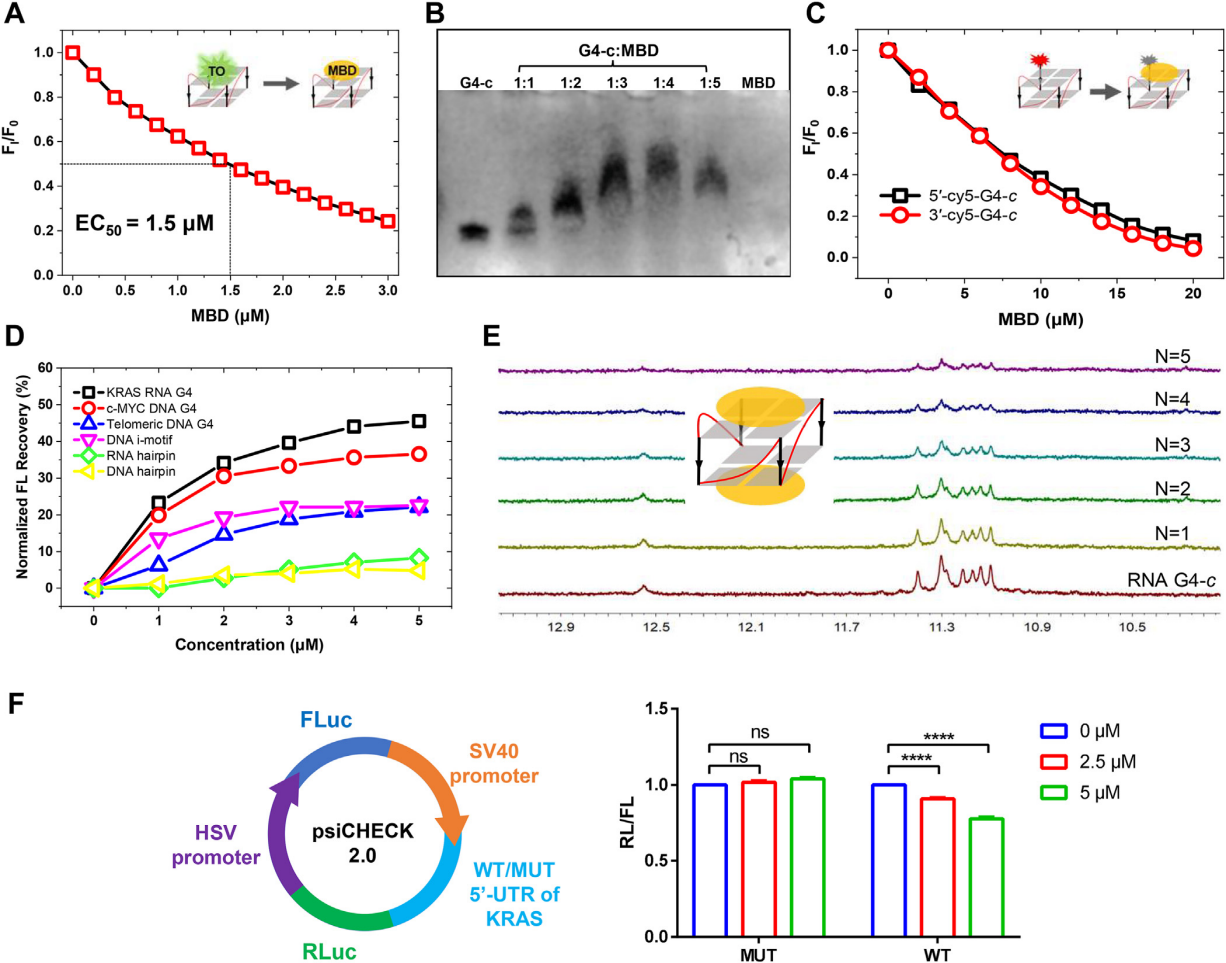
**Interaction of MBD with KRAS RG4.***A*, binding properties of MBD to KRAS RG4 determined by TO displacement assay, with F_0_ and F_1_ represented the fluorescence intensity of TO with G4 before and after the addition of MBD, respectively. *B*, binding properties of MBD to KRAS RG4 determined by EMSA, in which 1 to 5 M equivalents of MBD were added to KRAS RG4. *C*, fluorescence change of Cy5-labeled KRAS RG4 with the gradual addition of MBD, with F_0_ and F_1_ represented the fluorescence intensity of G4 before and after the addition, respectively. *D*, fluorescence recovery of Cy5-labeled KRAS RG4 with the gradual addition of different types of DNA or RNA sequences, after the fluorescence quenching of Cy5-labeled KRAS RG4 by MBD. *E*, effects of MBD on the guanine imino proton signals of KRAS RG4 by NMR assay, in which 1 to 5 M equivalents of MBD were added to KRAS RG4. *F*, relative ratio of Renilla luciferase activity to Firefly luciferase activity in psiCHECK-2 vector containing mutant or WT 5′-UTR of KRAS, without or with the presence of MBD. The data represent the mean ± SD (*n* = 3); ∗∗∗∗ for *p* < 0.0001 compared to the control group. RG4, RNA G-quadruplex; TO, thiazole orange.

The detailed binding activity of MBD to the representative KRAS RG4 motif (G4-c) was then decided by thiazole orange (TO) displacement assay. The fluorescence induced by TO in combination with nucleic acids would be quenched by the addition of competitive ligands (34). As shown in Figure 3*A*, TO fluorescence with KRAS RG4 distinctly decreased with the gradual addition of MBD. A strong binding affinity was demonstrated by the EC_50_ value of 1.5 μM. Whether MBD could effectively bind to KRAS RG4 was further determined by electrophoretic mobility shift assay, as reported before (35). After the addition of MBD, a band with lower mobility appeared other than KRAS RG4 alone. With the addition of 1 to 5 M equivalents of MBD to KRAS RG4, a regular delay of the band emerged and suggested that MBD could bind to KRAS RG4 to form a compound–G4 complex (Fig. 3*B*). In summary, MBD was a potential ligand to KRAS RG4 with high affinity.

To investigate the binding mode, CD spectra of MBD was collected in the region corresponding to the absorbance of the compound (350–650 nm, the absorption and fluorescence spectra were shown in Fig. S2). No induced CD signals could be observed upon the incubation of KRAS RG4, inferring the possibility of end stacking of MBD chromophore to KRAS RG4 (Fig. S3). We further performed fluorescence titration by gradual addition of the compound to G4s with individual Cy5 substitution at 5′-end or 3′-end regions. As shown in Figure 3*C*, the fluorescence of Cy5 at both regions was strongly affected, suggesting that MBD tended to bind onto both 5′ and 3′-end G-tetrads of KRAS RG4. Besides, right after the fluorescence quenching of Cy5-labeled KRAS RG4 by MBD, different types of DNA or RNA sequences were added gradually and fluorescence recovery was presented in Figure 3*D*. The most potent recovery occurred after the addition of KRAS RG4, whereas the addition of RNA hairpin and DNA hairpin hardly influenced the fluorescence signals. Therefore, MBD displayed selectivity to KRAS RG4 than other types of nucleic acid sequences. Moreover, ^1^H NMR spectra of KRAS RG4 interacting with MBD was recorded, and the results were displayed in Figure 3*E*. The signals of all of the guanine imino protons decreased gradually along with the addition of MBD, whereas no significant changes could be detected of the chemical shifts of guanine imino protons. ^1^H NMR spectra results proved that MBD could interact with two G-tetrads of KRAS RG4 through π-π stacking. To sum up, it was reasonable to hypothesize that MBD bound to both G-tetrads of KRAS RG4 with a 2:1 ratio.

A dual-luciferase reporter assay was carried out to illustrate the interaction of MBD and KRAS RG4 in cell model (26). PsiCHECK-2 vector was applied, in which Renilla luciferase (RL) is driven by the SV40 promoter and Firefly luciferase (FL) is driven by the HSV promoter. After the SV40 promoter, we inserted a WT 5′-UTR sequence of KRAS containing G4 structure, as well as a mutant one with no potential to form G4 structure (sequences shown in Table S2). As shown in Figure 3*F*, MBD decreased the relative activity of RL/FL in the WT KRAS RG4 group, but displayed no obvious effects in the mutant group. The above results proved that MBD repressed the translation of KRAS by mainly targeting the G4 structures in 5′-UTR sequence.

### Structural modification of MBD and their KRAS RG4 binding ability

**Figure 4:**
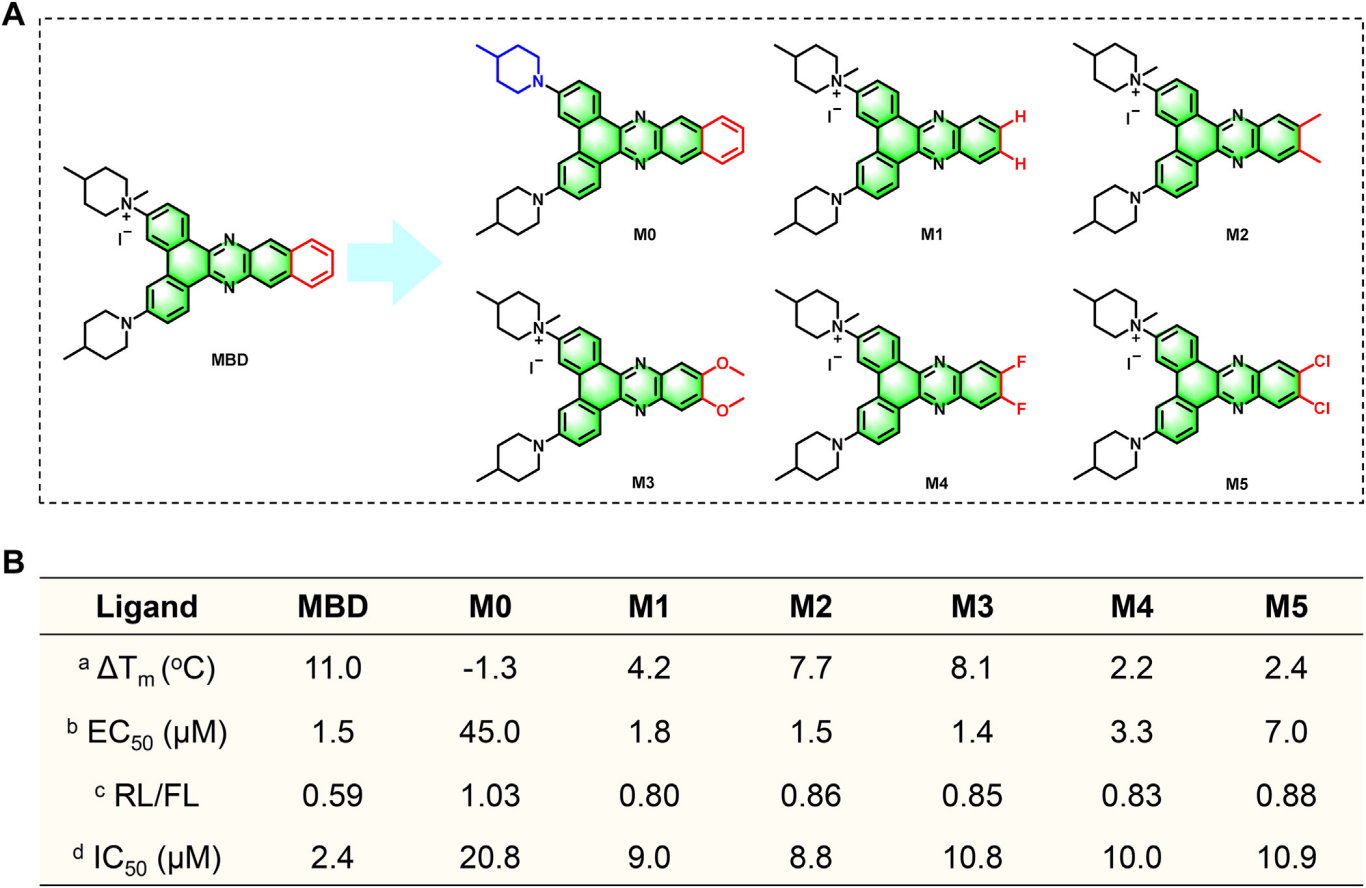
**Structural modification of MBD and their KRAS RG4 binding ability.***A*, the structures of the modified compounds (M0–M5). *B*, the binding properties of these compounds toward KRAS RG4, including Δ*T*_m_ by CD melting, EC_50_ by TO displacement, RL/FL by dual-luciferase reporter assay, and the IC_50_ of these compounds on A549/DDP cells by CCK8 assay. RG4, RNA G-quadruplex; FL, Firefly luciferase; RL, Renilla luciferase.

The above experiments demonstrated that MBD was indeed an effective KRAS RG4 binding ligand with a novel skeleton. Then, we showed interest in modifying this structure, hoping to acquire the structure-activity relationship regarding this skeleton. In this context, five compounds were designed and synthesized (Fig. 4*A*). After that, the stabilizing ability, binding affinity on KRAS RG4 as well as the cytotoxicity on A549/DDP cells were determined. As shown in Figure 4*B*, we observed that the remove of the positive charge (M0) completely erased its binding ability to KRAS RG4, leading to little cytotoxicity, which suggested that the existence of positive charge was an essential prerequisite. In addition, the remove of a phenyl group on the phenazine skeleton (M1) significantly reduced its binding to KRAS RG4 as well as the cytotoxicity, indicating this phenyl group played an important role in the recognition of KRAS RG4. Furthermore, we tried to introduce some electron-accepting or donating groups on the M1 scaffold for the sake of restoring the binding affinity. Then, we found that the introduction of electron-donating groups indeed enhanced the binding ability, especially evidenced by the increased Δ*T*_m_ values (M2 and M3), while the introduction of electron-accepting groups instead decreased the binding ability (M4 and M5). To be noted, the relative activity of RL/FL in the dual-luciferase reporter assay and the cytotoxicity on A549/DDP cells remained similar among the five modified compounds (M1–M5), which may be ascribed to the different uptakes of these compounds by cells. Therefore, MBD was identified as the most promising ligand for the further studies.

### Interaction of MBD with RG4s in A549/DDP cells

**Figure 5:**
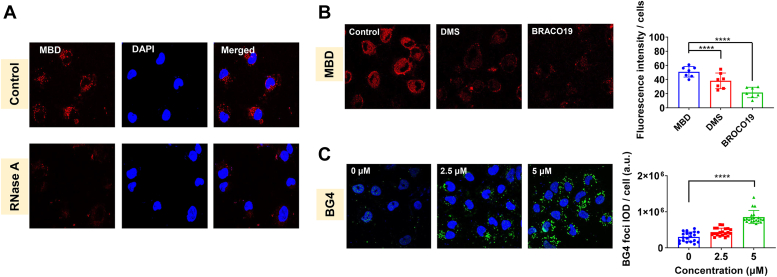
**Interaction of MBD with RG4s in A549/DDP cells.***A*, fluorescence images of fixed A549/DDP cells stained with MBD (*red*) and DAPI (*blue*) with or without the treatment of RNase A, displayed by confocal laser scanning microscope. *B*, fluorescence images of live A549/DDP cells stained with MBD (*red*), which were preincubated with DMS or BRACO19, displayed by confocal laser scanning microscope. Quantification of fluorescence intensity in each cell was also determined. *C*, immunofluorescence images of BG4 (*green*) foci in A549/DDP cells treated with MBD for 24 h, with the nuclei stained with DAPI (*blue*). Quantification of BG4 fluorescence intensity in each cell was also determined. ∗∗∗∗ for *p* < 0.0001 compared to the control group. RG4, RNA G-quadruplex.

Then, we examined the interaction behaviors of MBD in A549/DDP cells using the following experiments. Confocal laser scanning microscope was applied to determine the localization of MBD in the cells, and the results were shown in Figure 5*A*. A considerable amount of red fluorescence foci emerged around the cell nuclei, confirming that MBD could easily penetrate the cell membrane and accumulate in the cytoplasm. After RNase digestion, red fluorescence almost completely quenched, inferring that MBD probably targeted RNA G4s in the cytoplasm, rather than DNA G4s in the mitochondria.

In order to provide more evidence of MBD targeting RG4s, live A549/DDP cells were preincubated with dimethyl sulfate (DMS) or BRACO19 and then stained with MBD. DMS can methylate the N7 atoms of the guanine residues involved in Hoogsteen bonding, resulting in the prevention of the formation of G4s (36). Pretreatment of DMS decreased the red fluorescence, indicating that MBD selectively bound to G4s. Similar phenomena were observed in the cells pretreated with BRACO19, a typical G4 ligand, further proving the target of MBD as G4s (Fig. 5*B*).

RG4s were visualized by immunofluorescence assay with a well-known specific antibody against G4s (BG4 antibody) (37), in order to study whether MBD stabilized G4s in A549/DDP cells. After 24 h treatment, MBD triggered significant increase of BG4 intensity in the cytoplasm in a dose-dependent manner (Fig. 5*C*). To sum up, MBD could recognize and stabilize RG4 structures in A549/DDP cells.

### Signal transduction pathways induced by MBD in A549/DDP cells

**Figure 6:**
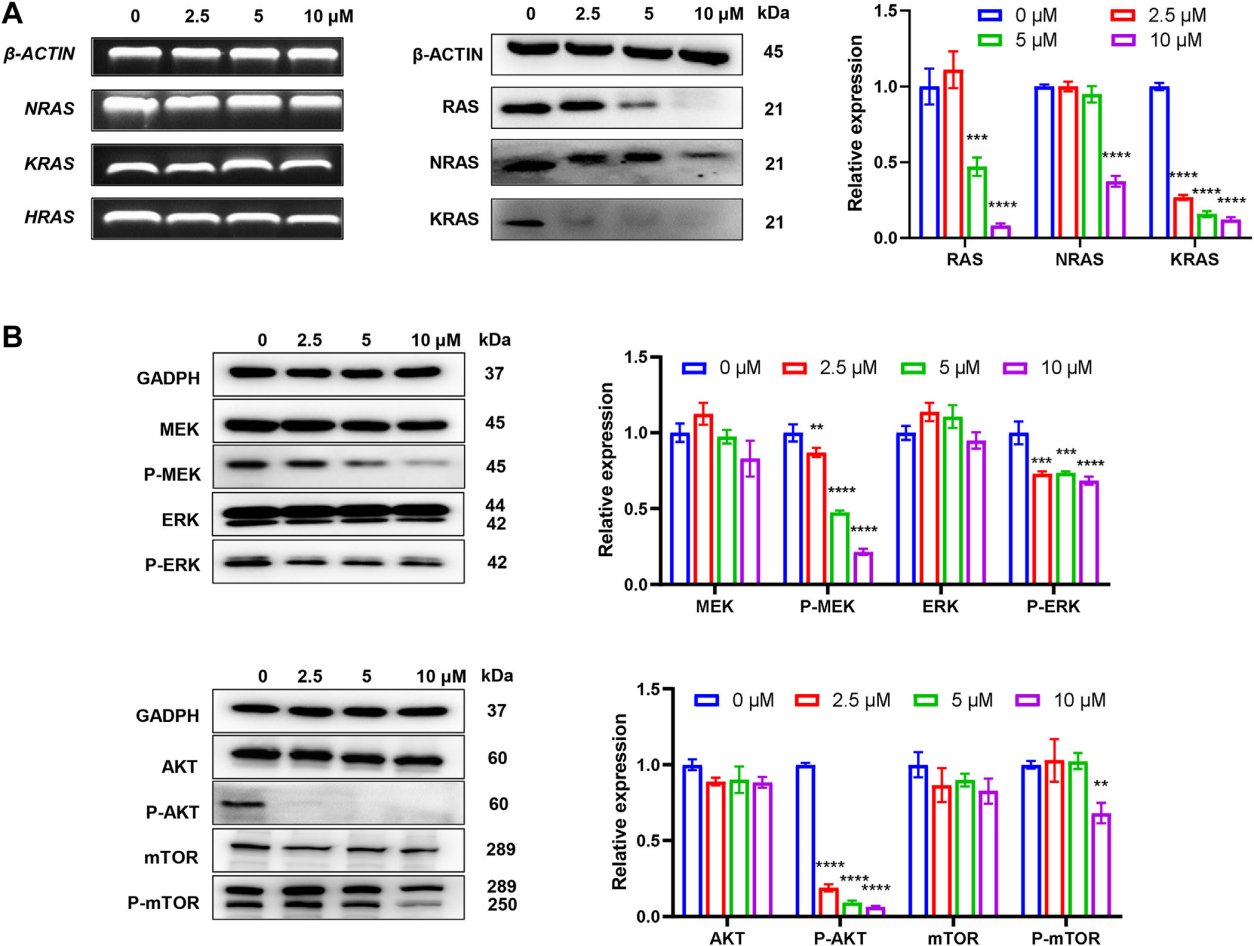
**Signal transduction pathways induced by MBD in A549/DDP cells.***A*, transcription and translation of RAS-related genes were detected by RT-PCR and Western blot in A549/DDP cells after 24 h drug treatment. β-Actin was used as the loading control. *B*, key proteins in MAPK and PI3K–AKT pathways were determined by Western blot in A549/DDP cells after 24 h drug treatment. GAPDH was used as the loading control. Image J (https://imagej.net) was used to calculate the relative expression levels. ∗∗ for *p* < 0.01, ∗∗∗ for *p* < 0.001, ∗∗∗∗ for *p* < 0.0001 compared to the control group.

As MBD was demonstrated to possibly bind and stabilize the KRAS RG4s in A549/DDP cells, we aimed to further investigate the anticancer mechanisms of MBD. RT-PCR and Western blot assays were first performed to investigate whether MBD repressed the transcription and translation of RAS-related genes. Results of all of the three RAS genes (KRAS, NRAS, and HRAS) were shown in Figure 6*A*. mRNA levels of the genes remained unchanged, but protein levels were definitely downregulated in A549/DDP cells treated by MBD. Expression of total RAS protein was decreased at the concentration of 5 μM. Decrease of NRAS expression occurred at a higher concentration of 10 μM (which may be ascribed to the existence of a RG4 in the 5′-UTR of NRAS mRNA), whereas decrease of KRAS expression occurred at a lower concentration of 2.5 μM. Therefore, MBD depressed the translation of RAS genes, and KRAS was the most vulnerable gene to be affected, inferring MBD could selectively target KRAS to a certain degree.

KRAS protein plays a critical role in the transduction of mitogenic signals, and a number of downstream effectors are reported and closely connected to tumorigenesis (38). MAPK signaling is a well-defined KRAS-mediated effector pathway, in which RAF phosphorylates mitogen-activated protein kinase kinase (MEK) and in turn phosphorylates extracellular signal-regulated kinase (ERK) (39). PI3K-AKT-mTOR signaling is another important KRAS-mediated effector pathway, in which PI3K converts PIP2 to PIP3, and PIP3 phosphorylates AKT and in turn phosphorylates mTOR (40). The effects of MBD on the above-mentioned signaling pathways in A549/DDP cells were examined by Western blot assay. MBD displayed little impact on the expression of total MEK, ERK, AKT, and mTOR, but the phosphorylation of MEK, ERK, AKT, and mTOR was significantly reduced by MBD in a dose-dependent manner (Fig. 6*B*). These findings ensured that MBD blocked both MAPK and PI3K–ATK signaling pathways in A549/DDP cells, in accordance with the KRAS repression.

**Figure 7:**
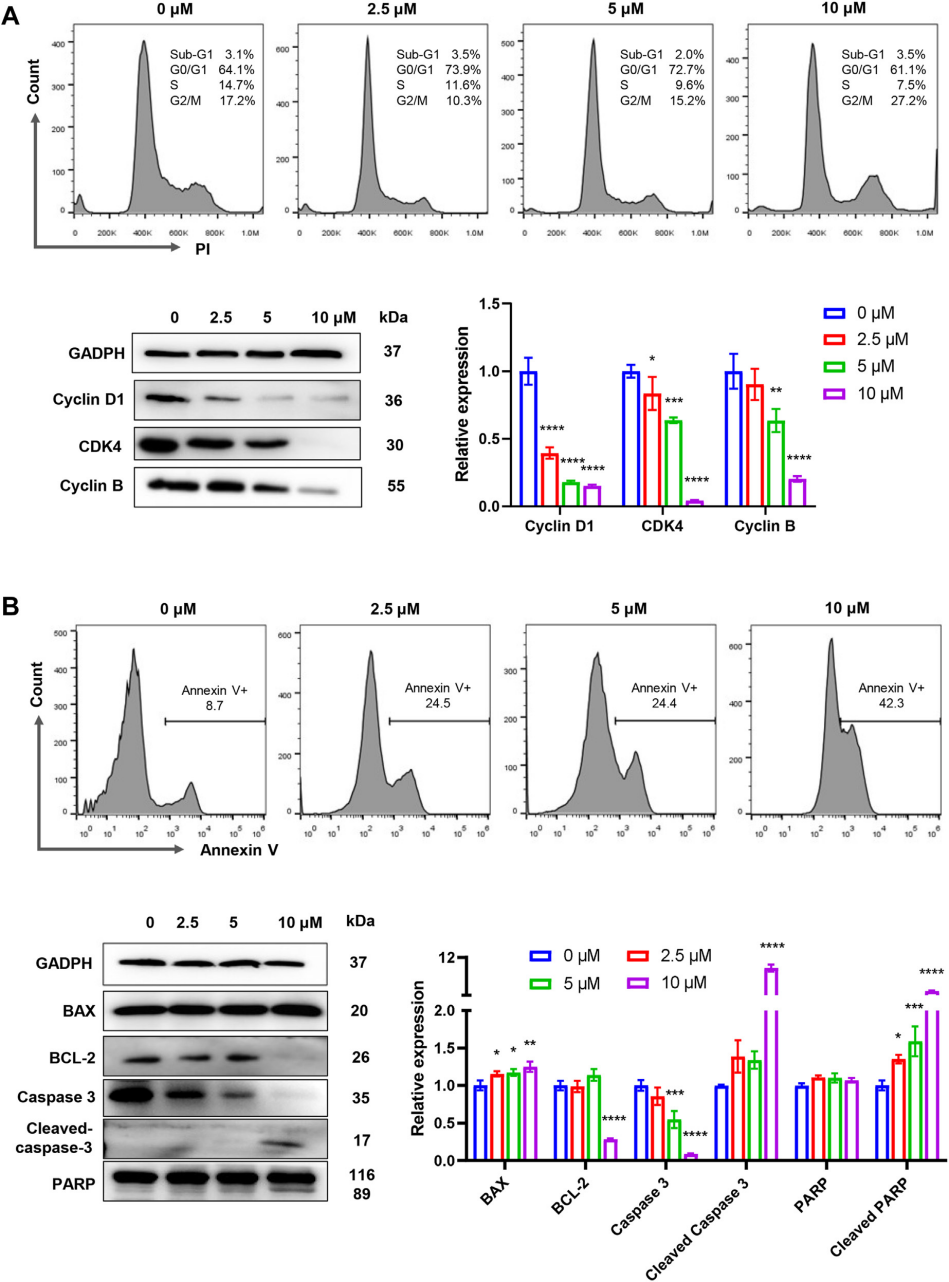
**Cell cycle arrest and apoptosis induced by MBD in A549/DDP cells.***A*, cell cycle was determined by PI staining and flow cytometry, whereas key proteins in cell cycle were determined by Western blot in A549/DDP cells after 24 h drug treatment. GAPDH was used as the loading control. *B*, apoptosis was determined by annexin V staining and flow cytometry, whereas key proteins in apoptosis were determined by Western blot in A549/DDP cells after 24 h drug treatment. GAPDH was used as the loading control. Image J was used to calculate the relative expression levels. ∗ for *p* < 0.05, ∗∗ for *p* < 0.01, ∗∗∗ for *p* < 0.001, and ∗∗∗∗ for *p* < 0.0001 compared to the control group.

MAPK and PI3K–AKT pathways are of great importance in the regulation of cell cycle, apoptosis, survival, and migration of tumor cells. Cell cycle was first to be determined by flow cytometry and the results were displayed in Figure 7*A*. MBD induced remarkable increase of G0/G1 population and decrease of G2/M population at the concentration of 2.5 μM, whereas G2/M arrest occurred from 2.5 μM to 10 μM in a dose-dependent manner. Cyclins and cyclin-dependent kinases are main regulatory proteins in cell cycle, where cyclin D1 and cyclin B are crucial for G0/G1 and G2/M phases, respectively (41, 42). Here, 2.5 μM MBD downregulated the expression of cyclin D1 and CDK4, while 10 μM MBD downregulated the expression of cyclin B. Overall, MBD induced G0/G1 arrest at lower concentrations and G2/M arrest at higher concentrations, probably due to the wide regulation of KRAS on a variety of pathways.

Apoptosis was then evaluated by flow cytometry in drug-treated A549/DDP cells. Only annexin V staining was performed, because MBD disturbs the fluorescence signals of propidium iodide (PI) and the cells cannot be effectively designated as PI− and PI+ populations. As shown in Figure 7*B*, a dose-dependent increase of apoptotic population (annexin V+) was recorded after the treatment of MBD. BAX and Bcl-2 permeabilize the outer mitochondrial membrane, while caspase-3 and poly (ADP-ribose) polymerase (PARP) are the final executioners in the intrinsic apoptosis pathway (43, 44). Here, MBD increased the expression of pro-apoptotic BAX and decreased the expression of anti-apoptotic Bcl-2. Full-length caspase-3 and PARP were cleaved, and the fragments of caspase-3 and PARP were detected, giving information of the occurrence and the pathway of MBD-induced apoptosis.

### Antitumor efficacy of MBD in A549/DDP cells *in vitro*

**Figure 8:**
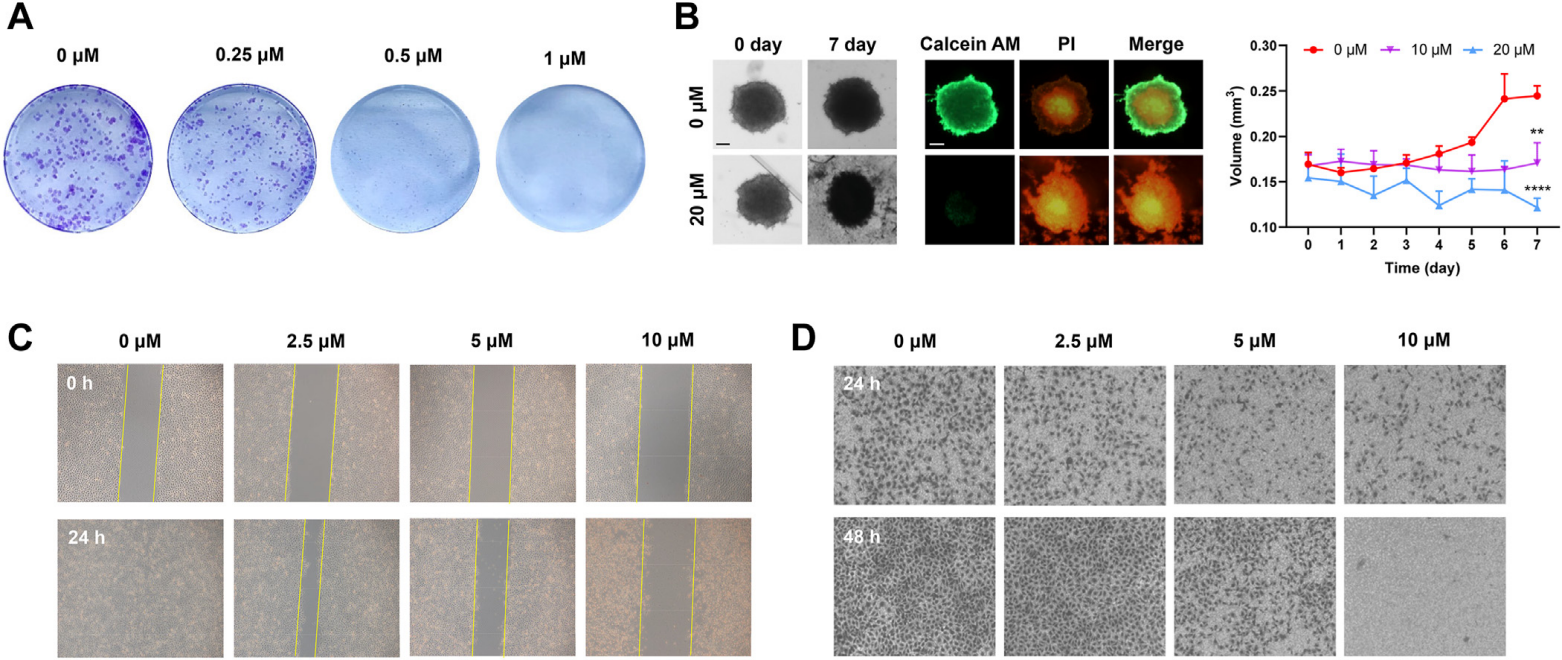
**Antitumor efficacy of MBD in A549/DDP cells *in vitro*.***A*, growth inhibition of A549/DDP cells treated by MBD for 7 days, displayed by colony formation assay. *B*, growth inhibition of A549/DDP cells treated by MBD for 7 days, displayed by MCTS assay. MCTS was observed and measured every day, and the viability was determined by calcein AM and PI dual staining on day 7. The scale bar represents 200 μm. *C*, inhibition of migration of A549/DDP cells treated by MBD for 24 h, displayed by wound healing assay. *D*, inhibition of migration of A549/DDP cells treated by MBD for 24 h and 48 h, displayed by transwell assay. MCTS, multicellular tumor spheroid.

The antitumor effects of MBD were first illustrated by colony formation assay, and the results were shown in Figure 8*A*. The numbers of purple spots decreased dramatically, suggesting the reduction of the clones of A549/DDP cells, especially with the treatment of high concentrations of MBD. After the assessment of cytotoxicity on 2D monolayer cells, we performed 3D multicellular tumor spheroid (MCTS) assay, which is a more approximate model to the condition of *in vivo* tumor growth (45). As shown in Figure 8*B*, the difference of tumor volumes between MBD group and control group was notable, and the viability of MCTS was further determined by calcein acetoxymethyl ester (AM) and PI dual staining. After the treatment of MBD for 7 days, cell death in MCTS was observed, as proved by the increase of PI-stained red fluorescence and the decrease of calcein AM-stained green fluorescence.

Suppressive effects of MBD on cell migration were first demonstrated by wound healing assay, and the results were shown in Figure 8*C*. The wound scrape of A549/DDP cells almost healed after 24 h in the control group, while MBD clearly inhibited the cell migration in a dose-dependent manner. Transwell assay is another experiment to determine the ability of cell invasion (46). A large number of A549/DDP cells migrated to the lower surface of the transwell membrane after 24 h and 48 h in the control group, whereas MBD distinctly decreased the numbers of migrating cells (Fig. 8*D*). In summary, MBD was verified to be a potent agent against tumor growth and metastasis.

### Antitumor efficacy of MBD in an A549/DDP xenograft tumor model of nude mice

**Figure 9:**
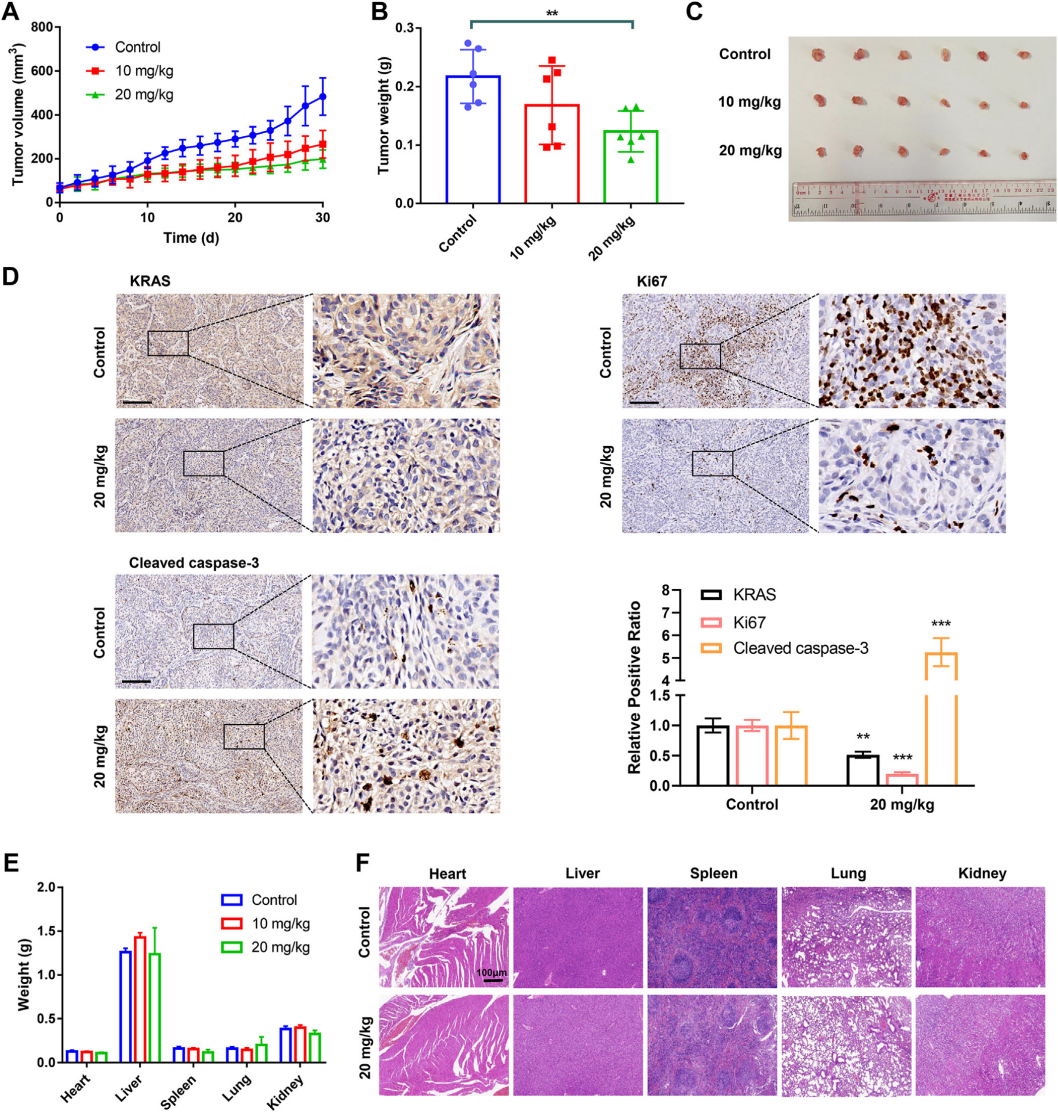
**Antitumor efficacy of MBD in a A549/DDP xenograft tumor model of nude mice.***A*, growth curves of the tumor volumes measured every other day until the end of the experiment. *B*, tumor weights measured at the end of the experiment. *C*, images of the excised tumors from all of the groups. *D*, expression of KRAS, Ki67, and cleaved caspase-3 in the tumor tissues determined by immunohistochemistry analysis. The scale bar represents 200 μm. For the quantification, tumors from three mice were detected in each group, with three sections for each tumor. Five areas were quantified for each section, and each area was 1 mm × 0.5 mm (about 3000 cells per area). *E*, weights of the major organs of the mice determined at the time of sacrifice. *F*, representative H & E staining images of the major organs of the mice. The scale bar represent 100 μm. The data represent the mean ± SD (n ≥ 5); ∗∗ for *p* < 0.01, ∗∗∗ for *p* < 0.001 compared to the control group.

Antitumor effects of MBD were evaluated *in vivo* in a nude mouse xenograft model inoculated with A549/DDP cells. The tumor-bearing mice were divided randomly into three groups and treated with saline (control), 10 mg/kg MBD and 20 mg/kg MBD every other day for 30 days. According to the tumor growth curves, tumor volumes were effectively suppressed by drug treatment, where 20 mg/kg MBD exerted more remarkable antitumor effects than 10 mg/kg MBD (Fig. 9*A*). The tumors were removed, weighed, and photographed at the end of the experiment, and the results were shown in Figure 9, *B* and *C*. MBD at 10 mg/kg and 20 mg/kg resulted in a noticeable reduction of tumor weights, with tumor growth inhibition of 23% and 43%, respectively, compared to the control group. Then, whether MBD could downregulate the expression of KRAS *in vivo* was discussed by immunohistochemistry assay. As shown in Figure 9*D*, KRAS expression of tumor tissues in MBD (20 mg/kg) group was obviously lower than in the control group, suggesting that the *in vivo* antitumor efficacy of MBD could be related to its specific target of KRAS. Furthermore, downregulation of Ki67 and upregulation of cleaved caspase-3 were observed after drug treatment, inferring that the antitumor effects were due to both decreased proliferation and increased cell death.

The mice in all of the groups appeared healthy without any visible signs of pain or discomfort. Body weights were recorded every other day, where only high dose of MBD (20 mg/kg) exhibited some weight loss (Fig. S4). We further harvested the major organs (hearts, livers, spleens, lungs, and kidneys) of the mice, and no significant differences in the weights of the major organs could be detected between the groups (Fig. 9*E*). Additionally, results of H&E staining verified that no serious structural or pathological changes occurred in the major organs after drug treatment, as shown in Figure 9*F*. All of the above data confirmed that MBD was a safe and effective antitumor agent against cisplatin-resistant NSCLC.

## Conclusions

5′-UTR of KRAS mRNA contains a G-rich sequence with the potential to form RG4 structures. Small molecules binding and stabilizing KRAS RG4s are determined with the ability of the downregulation of KRAS expression, leading to the suppression of tumor growth. However, only a few ligands have been discovered until now, and there is an urgent need to identify new types of ligands targeting KRAS RG4s. In this study, we hierarchically screened an in-house G4 ligand library, and obtained a lead compound, MBD, with the highest possibility and capability to target KRAS RG4s. The interaction studies found that MBD could bind to both G-tetrads of KRAS RG4 with a 2:1 ratio, displayed by CD, fluorescence, and NMR spectroscopy. The antitumor mechanisms of MBD were then investigated in A549/DDP cells, a cisplatin-resistant and KRAS-mutant NSCLC cell line. MBD recognized and stabilized RG4s in A549/DDP cells, and downregulated the translation of KRAS, rather than the transcription. MAPK and PI3K pathways, two representative KRAS downstream pathways, were blocked by MBD in a dose-dependent manner, resulting in the arrest of cell cycle and the occurrence of cell apoptosis. Suppression on tumor growth and metastasis was verified in A549/DDP cells *in vitro* by colony formation, MCTS formation, wound scrape and transwell assays. Efficacy and safety of MBD were eventually shown in a A549/DDP xenograft tumor model of nude mice. Certainly, there were some limitations for *in vivo* study, where 20 mg/kg MBD displayed moderate inhibition on tumor growth, as well as some toxicity to the mice with body weight decrease. Besides, there could be off-target effects *in vivo*, since MBD was possible to bind to other types of RG4s. Thus, structural optimization of the ligand, together with target identification and validation, should be performed in future in more detail. Although there are still some issues remained to be solved, this work gives a new choice of RG4-targeted agents for the treatment of KRAS-driven NSCLC.

## Experimental procedures

### Synthesis and characterization

The synthetic routes of the compounds were described in Figure 1 (MBD) and S1 (M1–M5): (a) 3,6-dibromophenanthrene-9,10-dione (5 mmol), 4-methylpiperidine (25 mmol), and appropriate amounts of K_2_CO_3_ (10 mmol) were mixed in a clean round-bottom flask, and then dimethyl sulfoxide (DMSO) (10 ml) were added. After that, the mixture was stirred under reflux for 5 h. After cooling, the mixture was poured into the icy water, and the precipitate was filtered to obtain a purple solid powder (intermediate 2). (b) Intermediate 2 (2.0 mmol) was mixed with 2,3-diaminonaphthalene (5.0 mmol), and 10 ml of anhydrous ethanol was added to fully dissolve the mixture. Three drops of acetic acid were added as a catalyst, and the mixture was stirred under reflux overnight. After cooling, the mixture was filtered to obtain a red solid powder (intermediate 3). The intermediates 4 to 8 were obtained according to the same procedure. (c) Intermediate 3 (1.5 mmol) was dissolved in 10 ml of CHCl_3_/MeOH (1:1), and excess CH_3_I (15 mmol) was added, and then the reaction was kept at 60 °C overnight. After cooling, the solvent was removed by rotary evaporator, and the crude product was further purified by chromatography with eluent of CH_2_Cl_2_/MeOH (20:1). Finally, a red solid powder (MBD) was obtained. The compounds M1 to M5 were obtained according to the same procedure. Their structures and purities were confirmed by ^1^H NMR, ^13^C NMR, HPLC, and high-resolution mass spectrometry (HRMS).

Intermediate 2: 95% yield. ^1^H NMR (600 MHz, CDCl_3_) δ 8.00 (d, *J* = 8.9 Hz, 2H), 7.14 (d, *J* = 2.4 Hz, 2H), 6.76 (d, *J* = 2.4 Hz, 1H), 6.75 (d, *J* = 2.4 Hz, 1H), 3.98 (d, *J* = 13.1 Hz, 4H), 3.00 (td, *J* = 12.8, 2.6 Hz, 4H), 1.81 (d, *J* = 12.6 Hz, 4H), 1.73 – 1.65 (m, 2H), 1.31 (ddd, *J* = 16.2, 12.6, 4.0 Hz, 4H), and 1.00 (d, *J* = 6.6 Hz, 6H). Mass spectrometry (MS) (electrospray ionization [ESI]) *m/z*: 403 [M + H]^+^.

Intermediate 3 (also termed M0): 80% yield. ^1^H NMR (600 MHz, CDCl_3_/CD_3_OD) δ 9.12 (d, *J* = 8.9 Hz, 2H), 8.75 (s, 2H), 8.12 (dd, *J* = 6.4, 3.2 Hz, 2H), 7.77 (s, 2H), 7.52 (dd, *J* = 6.5, 3.0 Hz, 2H), 7.32 (dd, *J* = 8.9, 1.6 Hz, 2H), 4.00 (d, *J* = 12.4 Hz, 4H), 2.99 (t, *J* = 11.7 Hz, 4H), 1.87 (d, *J* = 12.4 Hz, 4H), 1.73 – 1.62 (m, 2H), 1.46 (dd, *J* = 21.6, 11.4 Hz, 4H), and 1.05 (d, *J* = 6.5 Hz, 6H). MS (ESI) *m/z*: 525 [M + H]^+^.

Intermediate 4: 90% yield. ^1^H NMR (400 MHz, CDCl_3_) δ 9.18 (d, *J* = 9.0 Hz, 2H), 8.21 (dd, *J* = 6.4, 3.4 Hz, 2H), 7.84 (s, 2H), 7.72 (dd, *J* = 6.5, 3.4 Hz, 2H), 7.34 (dd, *J* = 9.0, 2.4 Hz, 2H), 3.98 (d, *J* = 12.8 Hz, 4H), 2.96 (td, *J* = 12.2, 2.6 Hz, 4H), 1.86 (d, *J* = 12.4 Hz, 4H), 1.71 – 1.56 (m, 2H), 1.46 (qd, *J* = 12.0, 3.5 Hz, 4H), and 1.03 (d, *J* = 6.4 Hz, 6H). MS (ESI) *m/z*: 475 [M + H]^+^.

Intermediate 5: 85% yield. ^1^H NMR (600 MHz, CDCl_3_) δ 9.15 (d, *J* = 8.9 Hz, 2H), 7.96 (s, 2H), 7.86 (s, 2H), 7.34 (d, *J* = 2.4 Hz, 2H), 3.98 – 3.93 (m, 4H), 2.95 (td, *J* = 12.3, 2.6 Hz, 4H), 2.52 (s, 6H), 1.85 (dd, *J* = 12.2, 1.9 Hz, 4H), 1.67 – 1.58 (m, 2H), 1.47 (qd, *J* = 12.1, 3.9 Hz, 4H), and 1.03 (d, *J* = 6.5 Hz, 6H). MS (ESI) *m/z*: 503 [M + H]^+^.

Intermediate 6: 60% yield. ^1^H NMR (600 MHz, CDCl_3_) δ 9.13 (d, *J* = 8.9 Hz, 2H), 7.90 (s, 2H), 7.49 (s, 2H), 7.36 (dd, *J* = 8.9, 2.4 Hz, 2H), 4.12 (s, 6H), 4.02 – 3.94 (m, 4H), 2.99 – 2.92 (m, 4H), 1.86 (dd, *J* = 12.9, 3.6 Hz, 4H), 1.66 – 1.62 (m, 2H), 1.51 – 1.46 (m, 4H), and 1.04 (d, *J* = 6.5 Hz, 6H). MS (ESI) *m/z*: 535 [M + H]^+^.

Intermediate 7: 80% yield. ^1^H NMR (600 MHz, chloroform-*d*) δ 9.03 (d, *J* = 8.9 Hz, 2H), 7.89 – 7.78 (m, 4H), 7.30 (dd, *J* = 8.9, 1.9 Hz, 2H), 3.98 (dt, *J* = 13.1, 3.6 Hz, 4H), 3.03 – 2.92 (m, 4H), 1.91 – 1.82 (m, 4H), 1.71 – 1.60 (m, 2H), 1.50 – 1.46 (m, 4H), and 1.04 (d, *J* = 6.5 Hz, 6H). MS (ESI) *m/z*: 511 [M + H]^+^.

Intermediate 8: 80% yield. ^1^H NMR (400 MHz, chloroform-*d*) δ 9.03 (d, *J* = 9.0 Hz, 2H), 8.24 (s, 2H), 7.81 (s, 2H), 7.30 (dd, *J* = 8.9, 2.2 Hz, 2H), 3.99 (dt, *J* = 12.8, 3.2 Hz, 4H), 3.05 – 2.95 (m, 4H), 1.91 – 1.83 (m, 4H), 1.71 – 1.61 (m, 2H), 1.52 – 1.45 (m, 4H), and 1.05 (d, *J* = 6.4 Hz, 6H). MS (ESI) *m/z*: 543 [M + H]^+^.

1,4-Dimethyl-1-(6-(4-methylpiperidin-1-yl)tribenzo[*a*,*c*,*i*]phenazin-3-yl)piperidin-1-ium iodide (MBD): 40% yield. ^1^H NMR (600 MHz, DMSO-*d*_6_) δ 9.36 (d, *J* = 9.0 Hz, 1H), 8.99 (d, *J* = 9.0 Hz, 1H), 8.90 (s, 1H), 8.85 (d, *J* = 2.5 Hz, 1H), 8.82 (s, 1H), 8.31 (dd, *J* = 9.1, 2.4 Hz, 1H), 8.27 – 8.22 (m, 2H), 7.95 (d, *J* = 2.4 Hz, 1H), 7.69 – 7.59 (m, 2H), 7.44 (dd, *J* = 9.1, 2.3 Hz, 1H), 4.98 (d, *J* = 13.4 Hz, 2H), 4.20 – 4.14 (m, 2H), 4.04 (t, *J* = 12.9 Hz, 2H), 3.69 (s, 3H), 2.94 (td, *J* = 12.5, 2.6 Hz, 2H), 2.02 (d, *J* = 14.7 Hz, 2H), 1.95 – 1.88 (m, 1H), 1.82 (d, *J* = 12.7 Hz, 2H), 1.71 – 1.63 (m, 1H), 1.42 – 1.24 (m, 4H), 1.00 (d, *J* = 6.6 Hz, 3H), and 0.86 (d, *J* = 6.5 Hz, 3H). ^13^C NMR (151 MHz, DMSO-*d*_6_) δ 153.56, 144.38, 143.70, 141.70, 139.20, 138.00, 134.65, 134.47, 133.52, 132.29, 131.07, 128.84, 128.69, 128.64, 128.04, 127.80, 127.53, 127.00, 126.68, 121.79, 120.43, 117.43, 117.32, 108.18, 62.74, 60.62, 48.22, 33.96, 30.71, 29.53, 28.63, 22.27, and 21.01. HRMS (ESI) *m/z*: calcd for C_37_H_39_N_4_^+^ 539.3169 [M-I]^+^, found 539.3184 [M-I]^+^.

1,4-Dimethyl-1-(6-(4-methylpiperidin-1-yl)dibenzo[*a*,*c*]phenazin-3-yl)piperidin-1-ium iodide (M1): 45% yield. ^1^H NMR (600 MHz, DMSO-*d*_6_) δ 9.39 (d, *J* = 9.0 Hz, 1H), 9.03 (d, *J* = 9.0 Hz, 1H), 8.93 (d, *J* = 2.3 Hz, 1H), 8.31 (dd, *J* = 9.1, 2.4 Hz, 1H), 8.29 (dd, *J* = 8.4, 1.1 Hz, 1H), 8.24 (dd, *J* = 8.4, 1.0 Hz, 1H), 8.05 (d, *J* = 2.2 Hz, 1H), 7.97 (ddd, *J* = 8.3, 6.8, 1.4 Hz, 1H), 7.92 (ddd, *J* = 8.2, 6.8, 1.4 Hz, 1H), 7.50 (dd, *J* = 9.1, 2.3 Hz, 1H), 4.99 (d, *J* = 13.0 Hz, 2H), 4.19 (d, *J* = 12.7 Hz, 2H), 4.04 (t, *J* = 12.9 Hz, 2H), 3.68 (s, 3H), 2.97 (t, *J* = 11.3 Hz, 2H), 2.00 (d, *J* = 14.1 Hz, 2H), 1.94 – 1.86 (m, 1H), 1.83 (d, *J* = 11.3 Hz, 2H), 1.72 – 1.63 (m, 1H), 1.39 – 1.28 (m, 4H), 1.01 (d, *J* = 6.6 Hz, 3H), and 0.84 (d, *J* = 6.5 Hz, 3H). ^13^C NMR (101 MHz, DMSO) δ 153.41, 144.02, 142.85, 142.69, 141.11, 140.05, 134.12, 132.14, 131.43, 130.83, 130.13, 129.64, 129.15, 128.51, 127.63, 121.60, 120.58, 117.68, 117.42, 108.06, 62.73, 60.66, 48.40, 33.98, 30.72, 29.50, 28.60, 22.27, and 20.97. HRMS (ESI) *m/z*: calcd for C_33_H_37_N_4_^+^ 489.3023 [M-I]^+^, found 489.3018 [M-I]^+^.

1-(11,12-Dimethyl-6-(4-methylpiperidin-1-yl)dibenzo[*a*,*c*]phenazin-3-yl)-1,4-dimethylpiperidin-1-ium iodide (M2): 45% yield. ^1^H NMR (600 MHz, DMSO-*d*_6_) δ 9.35 (d, *J* = 9.0 Hz, 1H), 8.99 (d, *J* = 9.0 Hz, 1H), 8.93 (d, *J* = 2.6 Hz, 1H), 8.31 (dd, *J* = 9.2, 2.5 Hz, 1H), 8.06 (d, *J* = 2.4 Hz, 1H), 7.92 (d, *J* = 14.3 Hz, 2H), 7.49 (dd, *J* = 9.1, 2.4 Hz, 1H), 4.99 (d, *J* = 12.8 Hz, 2H), 4.17 (d, *J* = 12.5 Hz, 2H), 4.04 (t, *J* = 13.2 Hz, 2H), 3.68 (s, 3H), 2.94 (td, *J* = 12.5, 2.7 Hz, 2H), 2.47 (s, 3H), 2.46 (s, 3H), 2.01 (d, *J* = 14.4 Hz, 2H), 1.96 – 1.87 (m, 1H), 1.83 (d, *J* = 12.5 Hz, 2H), 1.71 – 1.63 (m, 1H), 1.38 – 1.28 (m, 4H), 1.01 (d, *J* = 6.6 Hz, 3H), and 0.84 (d, *J* = 6.5 Hz, 3H). ^13^C NMR (126 MHz, DMSO) δ 153.12, 143.61, 142.07, 141.95, 141.70, 140.68, 140.18, 139.01, 133.71, 131.79, 131.06, 128.18, 127.76, 127.29, 121.41, 121.05, 117.77, 117.31, 108.08, 62.71, 60.69, 48.51, 34.00, 30.72, 29.51, 28.62, 22.29, 20.99, 20.54, and 20.38. HRMS (ESI) *m/z*: calcd for C_35_H_41_N_4_^+^ 517.3326 [M-I]^+^, found 517.3337 [M-I]^+^.

1-(11,12-Dimethoxy-6-(4-methylpiperidin-1-yl)dibenzo[*a*,*c*]phenazin-3-yl)-1,4-dimethylpiperidin-1-ium iodide (M3): 30% yield. ^1^H NMR (600 MHz, DMSO-*d*_6_) δ 9.33 (d, *J* = 9.0 Hz, 1H), 9.00 (d, *J* = 9.0 Hz, 1H), 8.94 (d, *J* = 2.5 Hz, 1H), 8.30 (dd, *J* = 9.2, 2.5 Hz, 1H), 8.08 (d, *J* = 2.4 Hz, 1H), 7.52 (dd, *J* = 9.2, 2.2 Hz, 1H), 7.50 (s, 1H), 7.44 (s, 1H), 5.00 (d, *J* = 13.3 Hz, 2H), 4.16 (d, *J* = 12.3 Hz, 2H), 4.08 – 4.00 (m, 8H), 3.67 (s, 3H), 2.95 (td, *J* = 12.5, 2.6 Hz, 2H), 2.01 (d, *J* = 14.4 Hz, 2H), 1.94 – 1.87 (m, 1H), 1.84 (d, *J* = 12.7 Hz, 2H), 1.69 – 1.63 (m, 1H), 1.42 – 1.29 (m, 4H), 1.01 (d, *J* = 6.5 Hz, 3H), and 0.85 (d, *J* = 6.5 Hz, 3H). ^13^C NMR (126 MHz, DMSO) δ 154.29, 153.41, 152.83, 143.08, 140.49, 140.33, 138.83, 137.07, 133.04, 131.36, 131.04, 127.74, 126.80, 121.42, 121.13, 118.00, 117.22, 108.05, 106.50, 106.23, 62.68, 60.70, 56.69, 56.67, 48.67, 34.06, 30.73, 29.50, 28.62, 22.30, and 20.98. HRMS (ESI) *m/z*: calcd for C_35_H_41_N_4_O_2_^+^ 549.3224 [M-I]^+^, found 549.3244 [M-I]^+^.

1-(11,12-Difluoro-6-(4-methylpiperidin-1-yl)dibenzo[*a*,*c*]phenazin-3-yl)-1,4-dimethylpiperidin-1-ium iodide (M4): 40% yield. ^1^H NMR (600 MHz, DMSO-*d*_6_) δ 9.13 (d, *J* = 9.0 Hz, 1H), 8.87 (d, *J* = 2.5 Hz, 1H), 8.78 (d, *J* = 9.0 Hz, 1H), 8.30 (dd, *J* = 9.2, 2.4 Hz, 1H), 8.04 (dd, *J* = 10.9, 8.5 Hz, 1H), 7.95 – 7.90 (m, 2H), 7.42 (dd, *J* = 9.2, 2.3 Hz, 1H), 5.01 (d, *J* = 13.4 Hz, 2H), 4.18 (d, *J* = 12.5 Hz, 2H), 4.06 (t, *J* = 13.3 Hz, 2H), 3.71 (s, 3H), 2.95 (td, *J* = 12.6, 2.7 Hz, 2H), 2.04 (d, *J* = 14.7 Hz, 2H), 1.96 – 1.88 (m, 1H), 1.85 (d, *J* = 11.7 Hz, 2H), 1.76 – 1.65 (m, 1H), 1.41 – 1.29 (m, 4H), 1.02 (d, *J* = 6.6 Hz, 3H), and 0.87 (d, *J* = 6.5 Hz, 3H). HRMS (ESI) *m/z*: calcd for C_33_H_35_F_2_N_4_^+^ 525.2824 [M-I]^+^, found 525.2829 [M-I]^+^.

1-(11,12-Dichloro-6-(4-methylpiperidin-1-yl)dibenzo[*a*,*c*]phenazin-3-yl)-1,4-dimethylpiperidin-1-ium iodide (M5): 40% yield. ^1^H NMR (600 MHz, DMSO-*d*_6_) δ 9.14 (d, *J* = 9.0 Hz, 1H), 8.85 (d, *J* = 2.5 Hz, 1H), 8.71 (d, *J* = 9.0 Hz, 1H), 8.32 (dd, *J* = 9.1, 2.4 Hz, 1H), 8.15 (s, 1H), 8.07 (s, 1H), 7.89 (d, *J* = 2.9 Hz, 1H), 7.39 (dd, *J* = 9.4, 2.2 Hz, 1H), 5.01 (d, *J* = 12.9 Hz, 2H), 4.18 (d, *J* = 12.5 Hz, 2H), 4.07 (t, *J* = 13.4 Hz, 2H), 3.70 (s, 3H), 2.96 (td, *J* = 12.5, 2.6 Hz, 2H), 2.05 (d, *J* = 14.6 Hz, 2H), 1.98 – 1.90 (m, 1H), 1.84 (d, *J* = 12.7 Hz, 2H), 1.74 – 1.65 (m, 1H), 1.45 – 1.26 (m, 4H), 1.02 (d, *J* = 6.6 Hz, 3H), and 0.88 (d, *J* = 6.5 Hz, 3H). ^13^C NMR (126 MHz, DMSO) δ 153.51, 144.40, 143.44, 141.20, 140.80, 139.44, 134.38, 133.70, 132.32, 132.13, 130.22, 129.93, 129.42, 128.59, 127.81, 121.71, 119.51, 117.40, 117.28, 107.62, 62.79, 60.69, 48.11, 33.97, 30.72, 29.56, 28.61, 22.28, and 21.04. ^13^C NMR (126 MHz, DMSO) δ 153.34, 144.16, 142.53, 140.05, 139.96, 139.76, 138.15, 138.06, 134.02, 131.96, 130.18, 128.30, 127.46, 121.56, 119.77, 117.40, 117.28, 114.90, 114.28, 107.58, 62.78, 60.70, 48.22, 33.97, 30.71, 29.51, 28.60, 22.27, and 21.01. HRMS (ESI) *m/z*: calcd for C_33_H_35_Cl_2_N_4_^+^ 557.2233 [M-I]^+^, found 557.2229 [M-I]^+^.

### Materials

All oligonucleotides (sequences shown in Table S1) were dissolved in Tris–HCl buffer, and the concentrations were confirmed by a NanoDrop 1000 spectrophotometer (Thermo Fisher Scientific) based on the absorbance at 260 nm. The oligonucleotides were heated at 95 °C for 5 min and cooled gradually to room temperature (RT), to ensure the formation of G4 structures, which were further confirmed by CD assay. Compounds were dissolved in DMSO at 10 mM, then aliquoted, and stored at −80 °C as stock solutions.

The authenticity of A549/DDP cell line (Procell) was validated using short tandem repeat profiling, and the cell line was tested free from *mycoplasma* contamination.

### CD spectroscopy

CD signals were recorded on a Chirascan CD spectrophotometer (Applied Photophysics), with the following parameters, 1 nm bandwidth, 1 nm step size, 0.5 s per point, in the wavelength range of 230 to 330 nm. CD melting signals were recorded at interval of 5 °C in the range of 25 to 95 °C with a heating rate of 1 °C/min. Concentrations of RNA samples and MBD were 2 μM and 10 μM, respectively. The buffer condition was as below: 10 mM Tris–HCl buffer containing 1 mM KCl (pH 7.4). *T*_m_ values were fitted and calculated by Origin 9.0 (https://www.originlab.com).

### Fluorescence spectroscopy

Fluorescence signals were recorded on a FluoroMax-4 spectrophotometer (HORIBA) with 2 mm × 10 mm path length at 3 nm excitation and emission slit widths. The buffer condition was as below: 10 mM Tris–HCl buffer containing 100 mM KCl (pH 7.4). For TO displacement assay, the sample solution containing 0.25 μM KRAS RG4 and 0.5 μM TO was first prepared in 700 μl buffer. Subsequently, small aliquots of the MBD stock solution (200 μM) were sequentially added to the above prepared sample in increments of 0.2 μM each time (0.7 μl each time). The excitation and emission wavelengths were set at 500 nm and 528 nm, respectively.

For fluorescence quenching assay, the sample solution containing 1 μM Cy5-labeled KRAS RG4 was first prepared in 700 μl buffer. Then, small aliquots of the MBD stock solution (1 mM) were sequentially added to the above prepared sample in increments of 2 μM each time (1.4 μl each time). After the fluorescence quenching, different types of unlabeled DNA or RNA samples at a stock concentration of 500 μM were added to the solution, in increments of 1 μM each time (1.4 μl each time), to complete the fluorescence recovery assay. The excitation and emission wavelengths were set at 625 nm and 668 nm, respectively.

### Electrophoretic mobility shift assay

KRAS RG4 (10 μM) and MBD (0–50 μM) were first coincubated and then separated on 15% native polyacrylamide gel in Tris-borate-EDTA buffer running buffer at 60 V for 2 h. The gel was stained by EB and image by GelView 1500 Pro (BLT).

### NMR spectroscopy

KRAS RG4 was diluted in potassium phosphate buffer containing 10% D_2_O at the concentration of 200 μM. Different concentrations of MBD were added, and ^1^H NMR spectra were recorded on a 600 MHz NMR spectrometer (Bruker) at 25 °C.

### Dual luciferase reporter assay

PsiCHECK-2 vector (Promega) was inserted with a WT or mutant 5′-UTR sequence of KRAS containing G4 structure (sequences shown in Table S2). After the transfection of the plasmids into HEK293 cells, MBD was added to the cells and incubated for 24 h. Luciferase activity was measured with Dual-luciferase Reporter Assay System (Promega), according to the supplier’s instruction, on a Synergy H1 multimode microplate reader (BioTek). RL activity was normalized by FL activity.

### CCK8 assay

Different types of cells were seeded in 96-well plates with the density of 5000 cells per well, and treated with MBD at different concentrations for 24 h. Then, the cells were incubated with 10% CCK8 solution for further 1 h, and the absorbance at 450 nm was recorded on a Synergy H1 multi-mode microplate reader (BioTek).

### Confocal laser scanning microscopy

A549/DDP cells were fixed with 4% paraformaldehyde for 20 min, and then stained with MBD (5 μM) and DAPI (1 μM) for 20 min. For RNase digesting assay, before stained with MBD, the cells were treated with Triton-X 100 (0.1%, 37 °C, 30 min) and RNase A (100 U/ml, 37 °C, 2 h). For displacement assay, live A549/DDP cells were incubated with 10 μM DMS or BRACO19 for 30 min and then added with 5 μM MBD for another 30 min. For all of the above experiments, images were captured by a LSM 880 laser scanning confocal microscope (Zeiss).

### Immunofluorescence assay

MBD-treated A549/DDP cells were fixed with 4% paraformaldehyde for 20 min at RT, permeabilized with 0.1% Triton-X for 30 min at 37 °C, and blocked with 5% goat serum for 3 h at 37 °C. Then, the cells were incubated with BG4 (50 ng/μl) for 4 h at 37 °C, and anti-FLAG antibody (1:400, #14793, CST) overnight at 4 °C. Finally, anti-rabbit Alexa 488–conjugated antibody (1:500, #4412, CST) and DAPI were added for 3 h at 37 °C. Images were captured by a LSM 880 laser scanning confocal microscope (Zeiss).

### RT-PCR assay

Total RNA of MBD-treated A549/DDP cells was extracted with Cell Total RNA Isolation Kit (Foregene), according to the supplier’s instruction. The concentration of RNA was quantitated by a NanoDrop 1000 spectrophotometer (Thermo Fisher Scientific). Reverse transcription was completed with Transcriptor First Strand complementary DNA Synthesis Kit (Roche), according to the supplier’s instruction, to harvest complementary DNA. Afterward, PCR was performed on a PCR device (Thermo Fisher Scientific), and the program for all genes included a denaturing cycle (95 °C for 5 min) and 28 PCR cycles (95 °C for 30 s, 58 °C for 30 s, and 72 °C for 40 s). Sequences of the PCR primers were listed in Table S3. PCR products were confirmed with agarose gel electrophoresis and SuperRed staining.

### Western blot assay

Total protein of MBD-treated A549/DDP cells was extracted with radio immunoprecipitation assay buffer containing protease inhibitors for 30 min at 4 °C. The concentration of protein was quantitated with Pierce BCA Protein Assay Kit (Thermo Fisher Scientific), according to the supplier’s instruction. 20 μg protein was separated by SDS-PAGE and transferred to polyvinylidene fluoride membrane (Bio-Rad). The membrane was blocked in 5% skim milk for 1 h at RT and incubated with the primary antibodies overnight at 4 °C and the peroxidase-conjugated secondary antibodies for 1 h at RT. Information of the antibodies was listed in Table S4. Finally, protein bands were detected by Enhanced Chemiluminescence Kit (Bio-Rad), and captured by an imaging system (CLiNX).

### Cell cycle analysis

MBD-treated A549/DDP cells were collected and stained by Cell Cycle Assay Kit (Dojindo), according to the supplier’s instruction. Cell cycle signals were recorded by an Attune N x T Flow Cytometer (Thermo Fisher Scientific).

### Apoptosis analysis

MBD-treated A549/DDP cells were collected and stained by annexin V-633 Apoptosis Detection Kit (Dojindo), according to the supplier’s instruction. Apoptosis signals were recorded by an Attune N x T Flow Cytometer (Thermo Fisher Scientific).

### Colony formation assay

A549/DDP cells were cultured in the 6-well plate with the density of 500 cells/well and treated by MBD for 7 days. Then, the cells were fixed with 4% paraformaldehyde and stained with crystal violet. Images were captured after rinsing the plate with PBS.

### MCTS formation assay

A549/DDP cells were cultured in the 96-well round-bottom ultralow attachment plate (Corning) with the density of 2000 cells/well. MCTS aggregates were formed after 5 days, and then treated by MBD for 7 days. Viability of MCTS was determined by calcein AM/PI Double Staining Kit (Dojindo), according to the supplier’s instruction.

### Wound scrape assay

A549/DDP cells were cultured in the 6-well plate until a confluent single layer of the cells was formed. A vertical line on the bottom of the plate was produced to from a small wound area. Then, MBD was diluted in serum-free medium and added for 24 h. Images were captured after rinsing the plate with PBS.

### Transwell assay

Cell invasion chambers with membranes of 8-μm pores (Corning) were placed in the 24-well plate. A549/DDP cells in serum-free medium were added to the upper compartment, and medium containing 15% fetal bovine serum was added to the lower compartment. After treated by MBD for 24 h and 48 h, cells attached to the lower surface of the membrane were fixed with 4% paraformaldehyde, stained with crystal violet, and finally photographed.

### *In vivo* antitumor study

Four-week-old male BALB/c nude mice were housed in a specific pathogen-free condition with a 12 h light/dark cycle and fed with water and food *ad libitum*. Each mouse was injected subcutaneously to the right flank with 1 × 10^7^ A549/DDP cells mixed with Matrigel. After tumor volumes reached approximate 50 mm^3^, the mice were randomly divided into three groups, and treated with saline, 10 mg/kg MBD and 20 mg/kg MBD every other day. Tumor sizes and body weights were measured every other day, and tumor volumes were calculated as 1/2 × length × width^2^. After drug treatment for 30 days, the mice were sacrificed, and the tumors and the major organs (hearts, livers, spleens, lungs, and kidneys) were removed, photographed and weighed. The major organs were fixed with 4% paraformaldehyde for the following H&E staining. KRAS expression in the tumor samples was further evaluated by immunohistochemistry analysis. The animal experiments were approved by the Animal Ethics Committee of Shenzhen University.

## Statistics

The data were plotted and expressed as the mean ± SD, for at least three independently performed experiments. The data were statistically analyzed using one-way ANOVA with Tukey’s *post hoc* test, and the value of *p* < 0.05 was statistically significant.

## Data availability

Data are available from the authors upon request.

## Supporting information

This article contains supporting information.

## Conflict of interest

The authors declare that they have no conflicts of interest with the contents of this article.
