# Supplementary material for: Discovery of a tribenzophenazine analog for binding to the KRAS mRNA G-quadruplex structures in the cisplatin-resistant non–small cell lung cancer
Source: J Biol Chem. 2025 Jan 8;301(2):108164. doi: 10.1016/j.jbc.2025.108164 (PMC11847542; doi:10.1016/j.jbc.2025.108164)
Supplement: Supplementary Data [file mmc1.docx]

**Supporting Information**

**Discovery of a tribenzo****phenazine analog for binding to the KRAS mRNA G-quadruplex structures in the cisplatin-resistant non-small cell lung cancer**

Xiao-Dong Wang^†^, Jia-Hong Lin^†^, Ming-Hao Hu*

Nation-Regional Engineering Lab for Synthetic Biology of Medicine, International Cancer Center, School of Pharmacy, Shenzhen University Medical School, Shenzhen 518060, China

^†^ These authors contributed equally.

* Corresponding author, humhao1229@szu.edu.cn

**Table of Contents:**

| **Fig. S1.** The synthetic route of **M1** to **M5** | 2 |
| --- | --- |
| **Fig. S2.** The absorption and emission spectra of **MBD** in Tris-HCl buffer | 2 |
| **Fig. S3.** CD spectra of **MBD** with or without KRAS RG4 | 3 |
| **Fig. S4.** Body weights of the mice measured during the experiment | 3 |
| **Table S1.** Sequences of the oligonucleotides used in this study | 4 |
| **Table S2.** Inserted sequences to the dual-luciferase reporter plasmid | 4 |
| **Table S3.** Primers used in RT-PCR assay | 4 |
| **Table S4.** Antibodies used in western blot assay | 5 |
| **Fig. S5–28.** ^1^H NMR, ^13^C NMR, HRMS and HPLC spectra of the synthesized compounds | 6 |





**Fig. S1.** The synthetic route of **M1** to **M5**


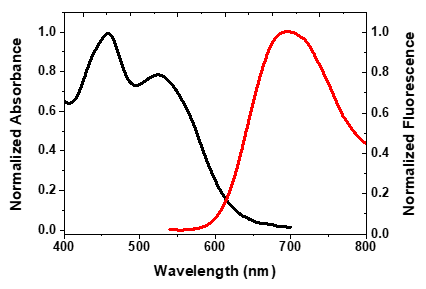


**Fig. S2.** The absorption and emission spectra of **MBD** in Tris-HCl buffer


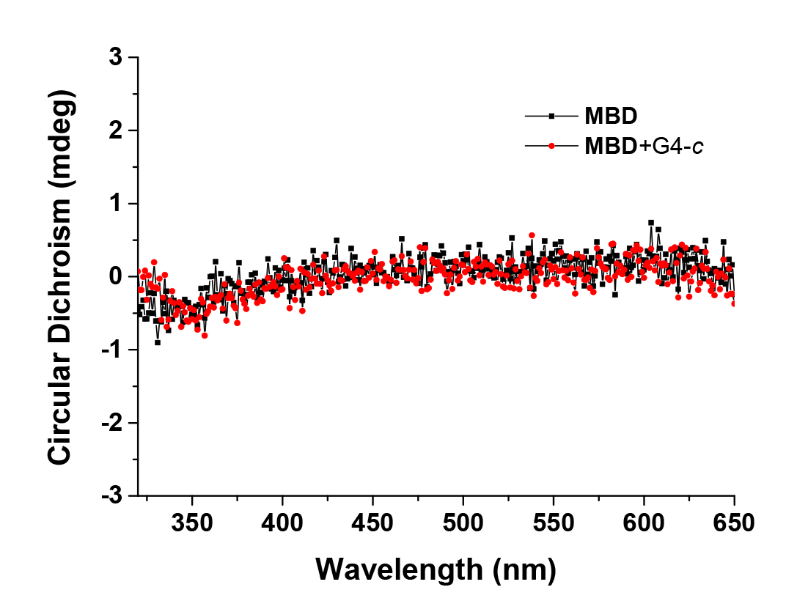


**Fig. S3.** CD spectra of **MBD** with or without KRAS RG4, in which no induced CD signal was observed, suggesting end-stacking of **MBD** to KRAS RG4.


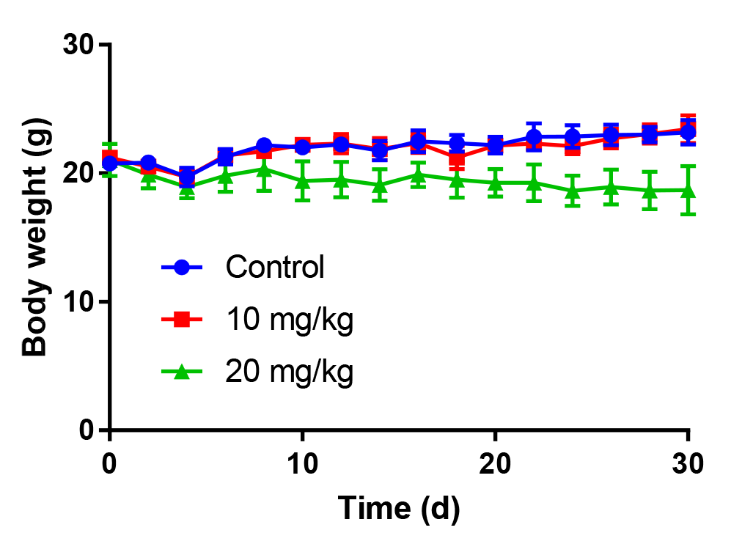


**Fig. S4.** Body weights of the mice measured every other day until the end of the experiment. The data represent the mean ± SE (n≥5).

**Table S1.** Sequences of the oligonucleotides used in this study

| Name | Sequence (5’→3’) |
| --- | --- |
| KRAS mRNA G4-a | GCGGCGGCGGAGGCAGCA |
| KRAS mRNA G4-b | GGCGGCGGCAGUGGCGGCGG |
| KRAS mRNA G4-c | AAGGUGGCGGCGGCUCG |
| c-myc DNA G4 | TGAGGGTGGGTAGGGTGGGTAA |
| Telomeric DNA G4 | AGGGTTAGGGTTAGGGTTAGGG |
| DNA i-motif | TTTTTTTTTTCCCTAACCCTAACCCTAACCC |
| RNA hairpin | GGCCGCCGCAGUGGCGGCGG |
| DNA hairpin | CGCGCGCGTTTTCGCGCGCG |

**Table S2.** Inserted sequences to the dual-luciferase reporter plasmid.

| Name | Sequence (5’→3’) |
| --- | --- |
| KRAS 5’-UTR  wild type | TCCTAGGCGGCGGCCGCGGCGGCGGAGGCAGCAGCGGCGGCGGCAGTGGCGGCGGCGAAGGTGGCGGCGGCTCGGCCAGTACTCCCGGCCCCCGCCATTTCGGACTGGGAGCGAGCGCGGCGCAGGCACTGAAGGCGGCGGCGGGGCCAGAGGCTCAGCGGCTCCCAGGTGCGGGAGAGAGGCCTGCTGAAA |
| KRAS 5’-UTR  mutant | TCCTAGGCGGCGGCCGCAACAACAAAAACAGCAGCAACAACAACAATAACAACAACGAAAATAACAACAACTCGGCCAGTACTCCCGGCCCCCGCCATTTCGGACTGGGAGCGAGCGCGGCGCAGGCACTGAAGGCGGCGGCGGGGCCAGAGGCTCAGCGGCTCCCAGGTGCGGGAGAGAGGCCTGCTGAAA |

**Table S3.** Primers used in RT-PCR assay.

| Gene | Forward primer (5’→3’) | Reverse primer (5’→3’) |
| --- | --- | --- |
| β-actin | GCATCCTGTCGGCAATGC | GTTGCTATCCAGGCTGTGC |
| KRAS | CGAATATGATCCAACAATAGAG | ATGTACTGGTCCCTCATT |
| NRAS | CCTATACAATGTATGTAATTTGTTTCC | CAATGCACCAAAGTTTTACAATATTTGAAC |
| HRAS | TTTGTGGACGAGTATGATCCCA | TGCTCCCTGTACTGATGGATG |

**Table S4.** Antibodies used in western blot assay.

| Antibody | Source | Cat# | Dilution |
| --- | --- | --- | --- |
| RAS | Abcam | ab55391 | 1:1000 |
| NRAS | Abcam | ab198820 | 1:1000 |
| KRAS | Abcam | ab275876 | 1:1000 |
| MEK | CST | 8727 | 1:1000 |
| P-MEK | CST | 9154 | 1:1000 |
| ERK | CST | 4695 | 1:1000 |
| P-ERK | CST | 4370 | 1:1000 |
| AKT | CST | 9272 | 1:1000 |
| P-AKT | CST | 9271 | 1:1000 |
| mTOR | CST | 2983 | 1:1000 |
| P-mTOR | CST | 5536 | 1:1000 |
| Cyclin D1 | CST | 2978 | 1:1000 |
| CDK4 | CST | 12790 | 1:1000 |
| Cyclin B | CST | 12231 | 1:1000 |
| BAX | CST | 5023 | 1:1000 |
| BCL-2 | CST | 4223 | 1:1000 |
| Caspase-3 | CST | 14220 | 1:1000 |
| Cleaved Caspase-3 | CST | 9664 | 1:1000 |
| PARP | CST | 9532 | 1:1000 |
| GAPDH | CST | 5174 | 1:1000 |
| β-ACTIN | CST | 4970 | 1:1000 |


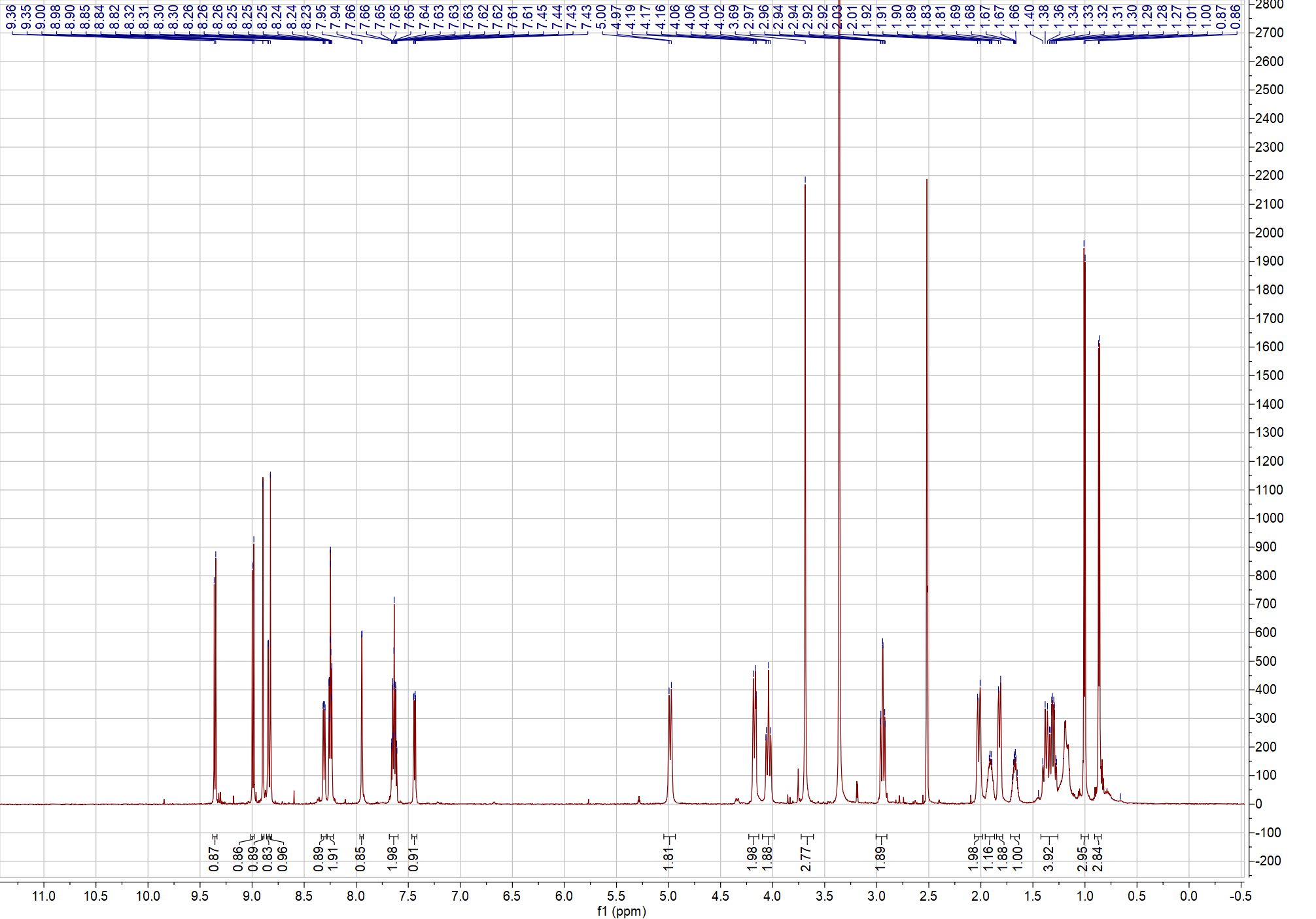


**Fig. S5.** ^1^H NMR spectrum of **MBD**


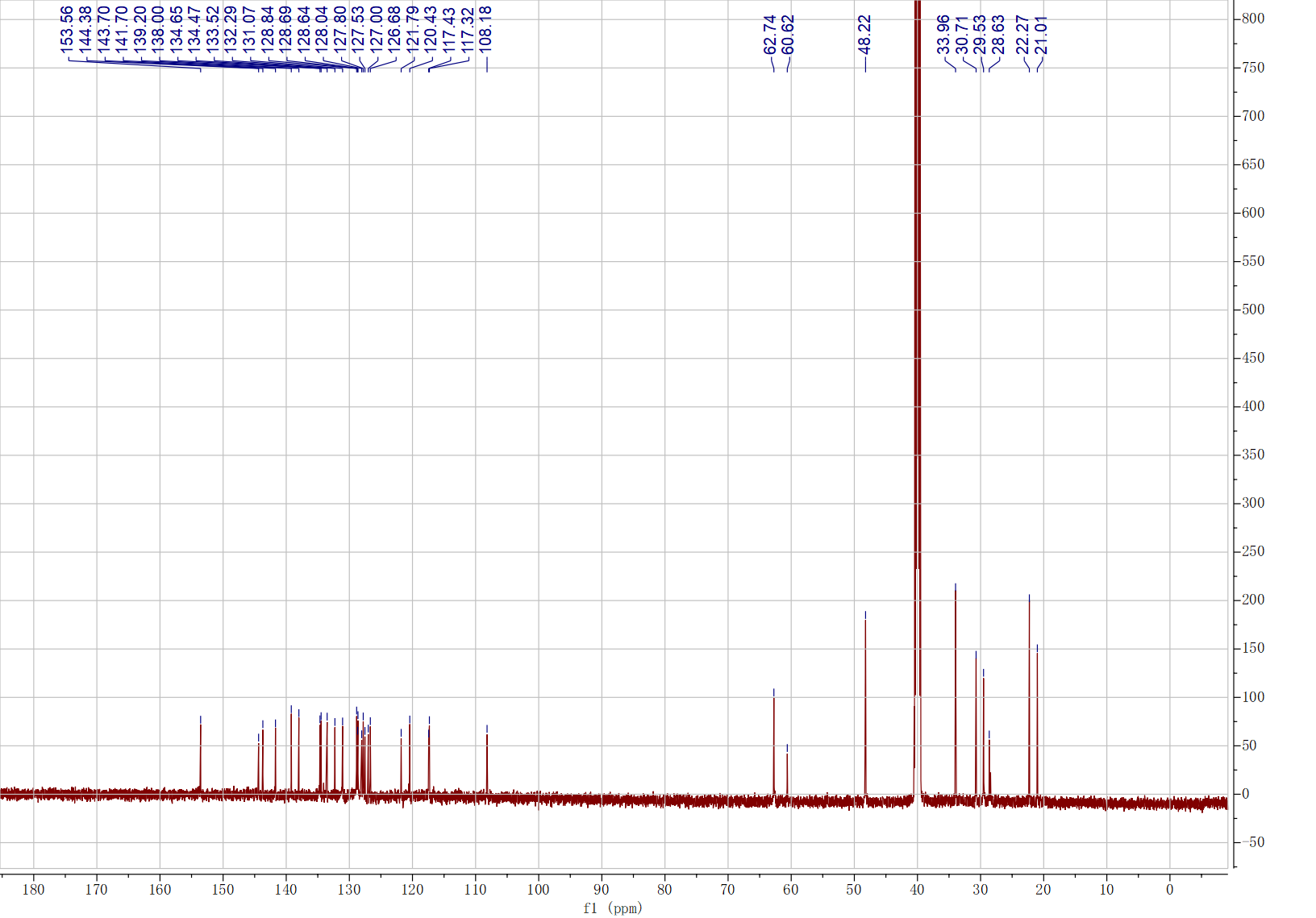


**Fig. S6.** ^13^C NMR spectrum of **MBD**

**Fig. S7.** HRMS spectrum of **MBD**


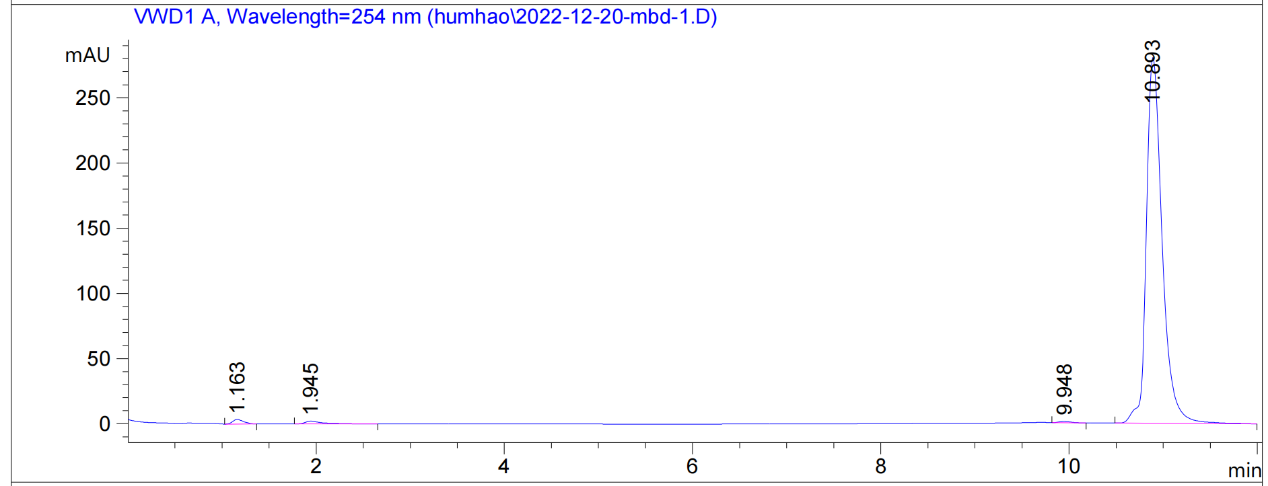


**Fig. S8.** HPLC spectrum of **MBD**


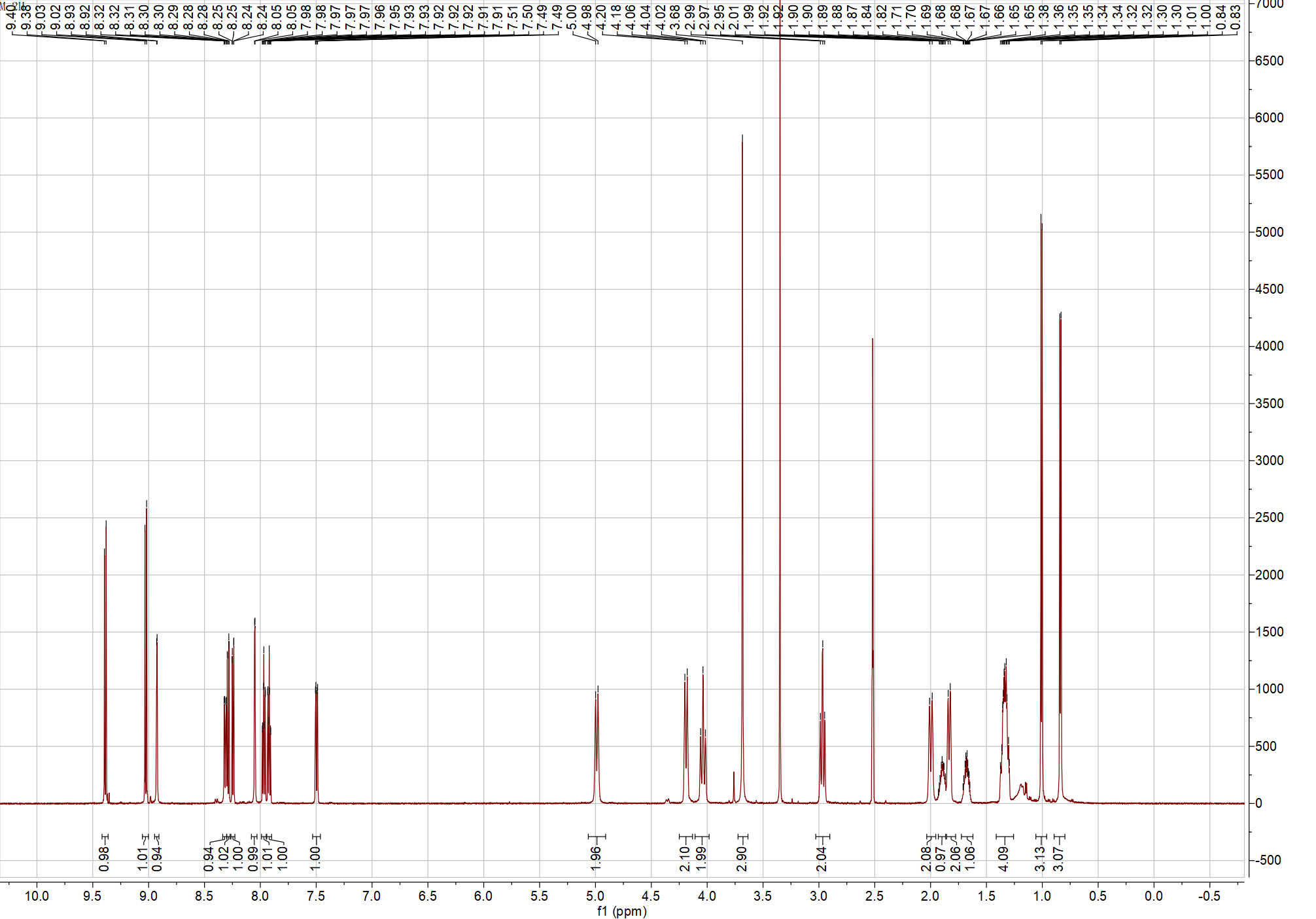


**Fig. S9.** ^1^H NMR spectrum of **M1**


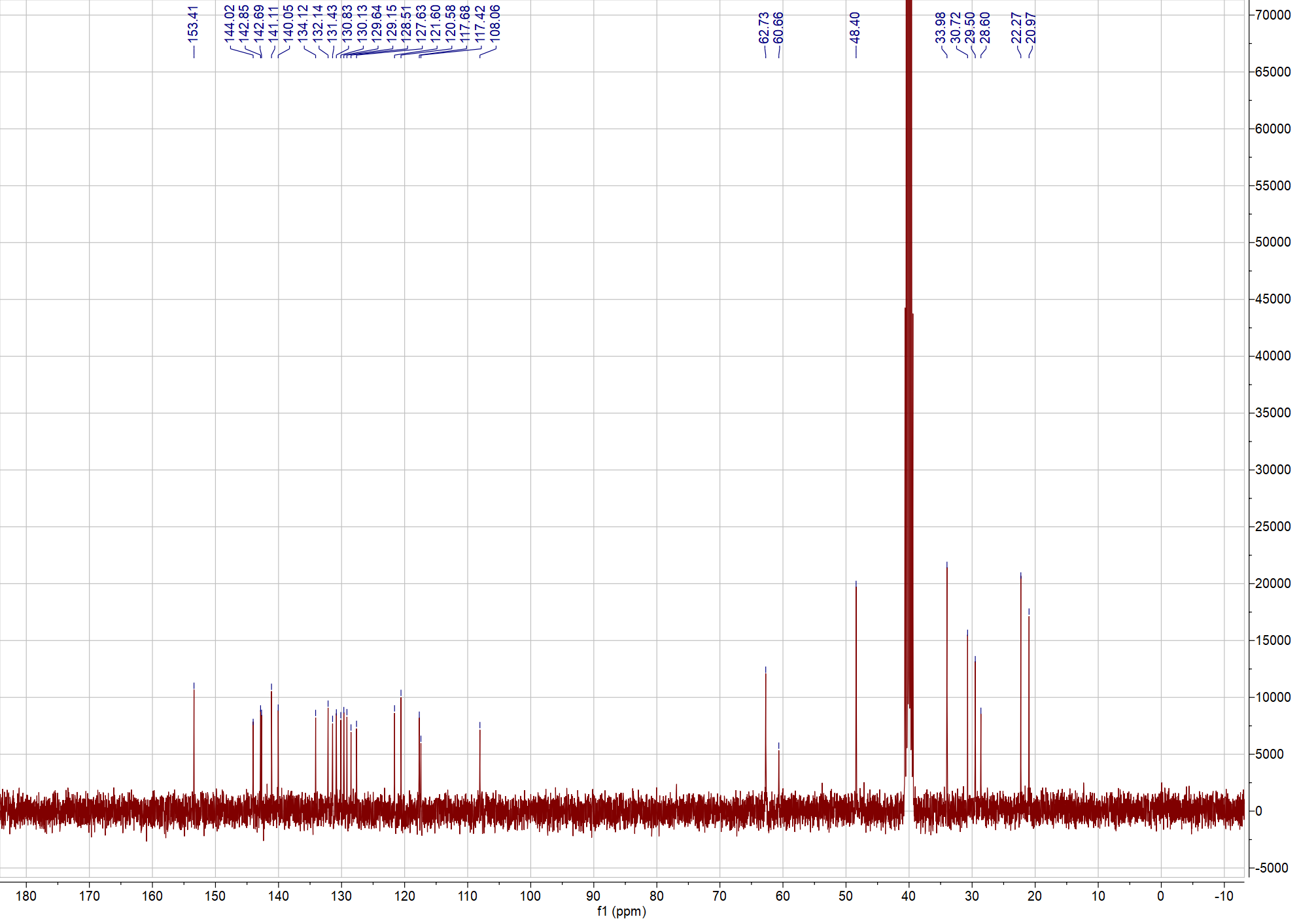


**Fig. S10.** ^13^C NMR spectrum of **M1**

**Fig. S11.** HRMS spectrum of **M1**

**
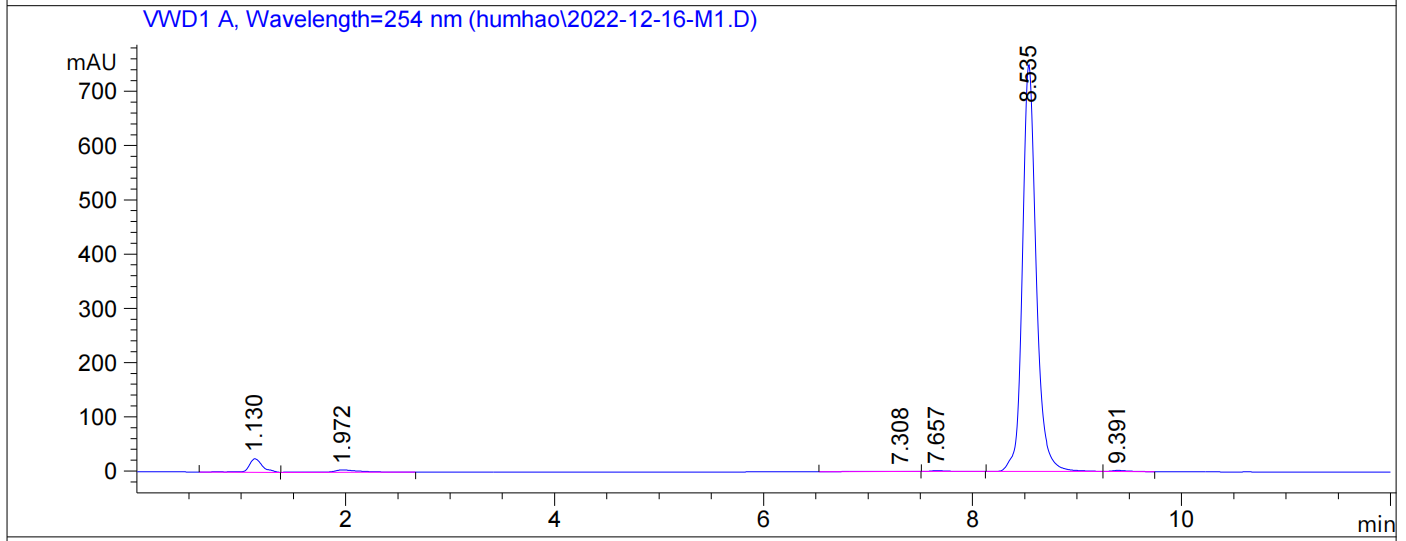
**

**Fig. S12.** HPLC spectrum of **M1**


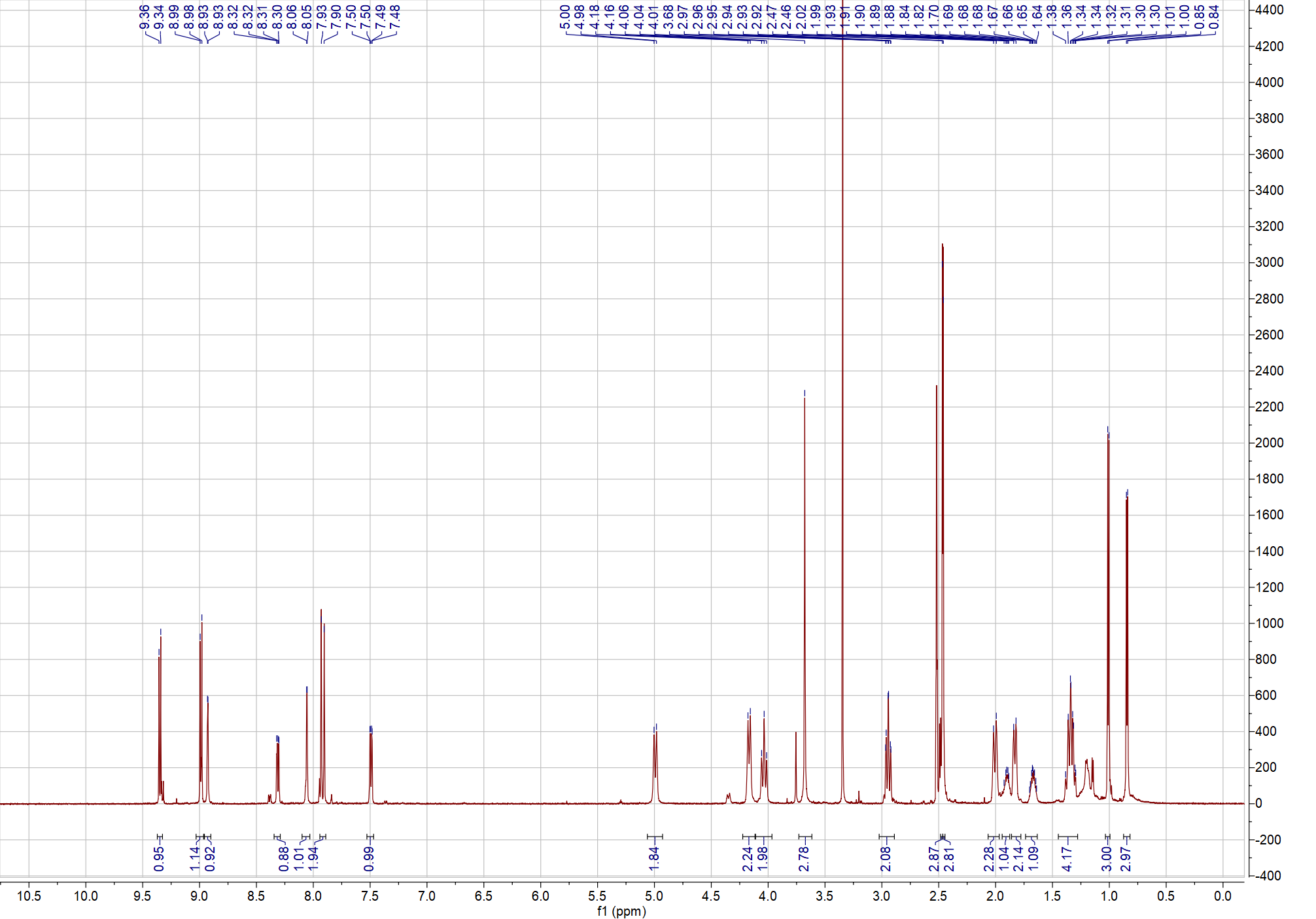


**Fig. S13.** ^1^H NMR spectrum of **M2**

**
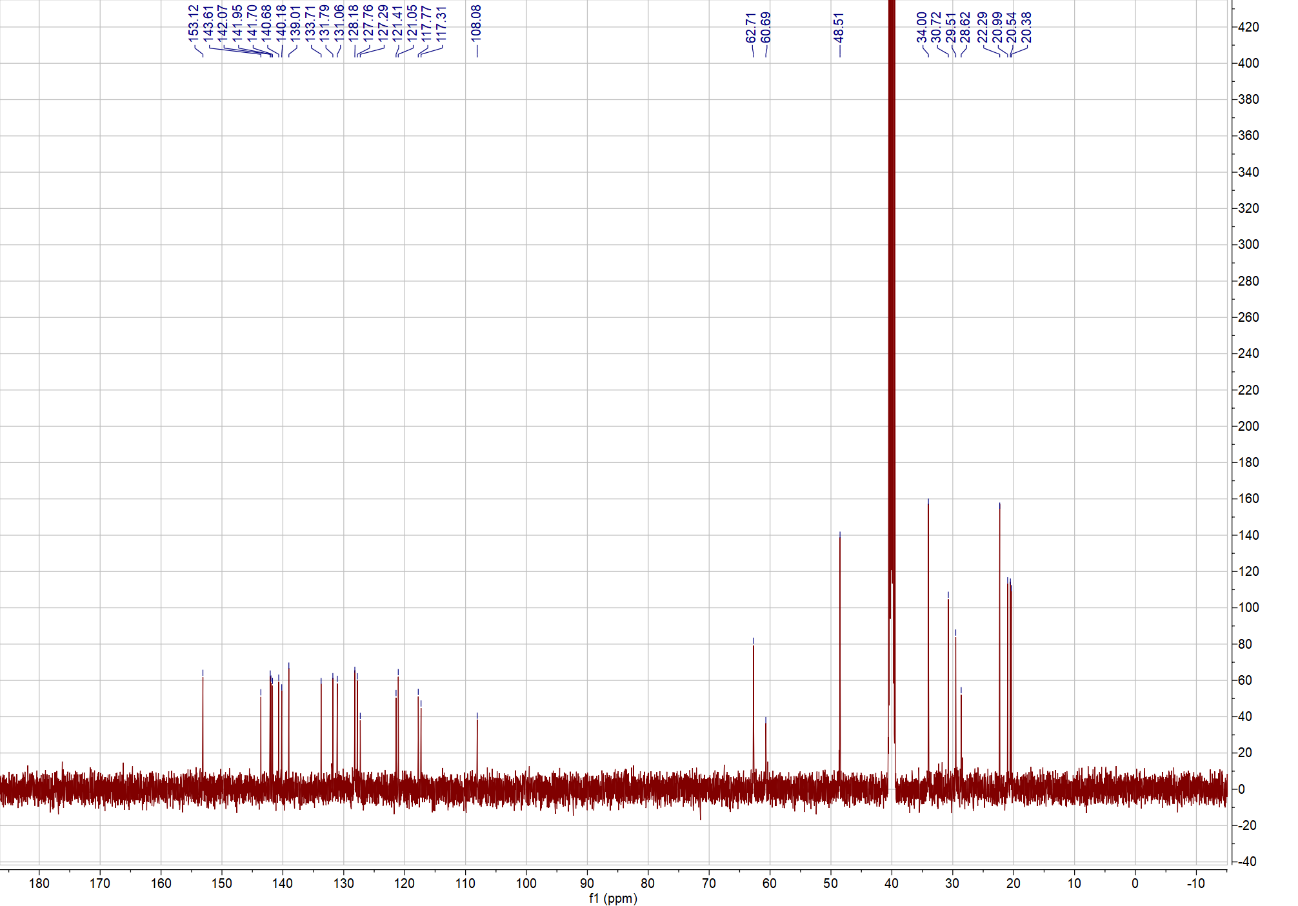
**

**Fig. S14.** ^13^C NMR spectrum of **M2**

**Fig. S15.** HRMS spectrum of **M2**

**
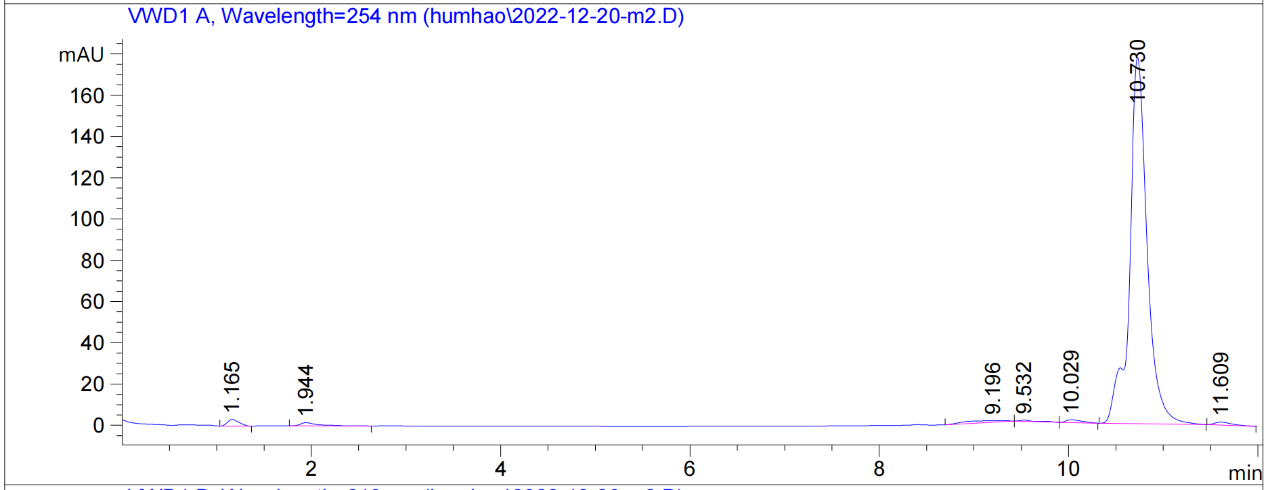
**

**Fig. S16.** HPLC spectrum of **M2**


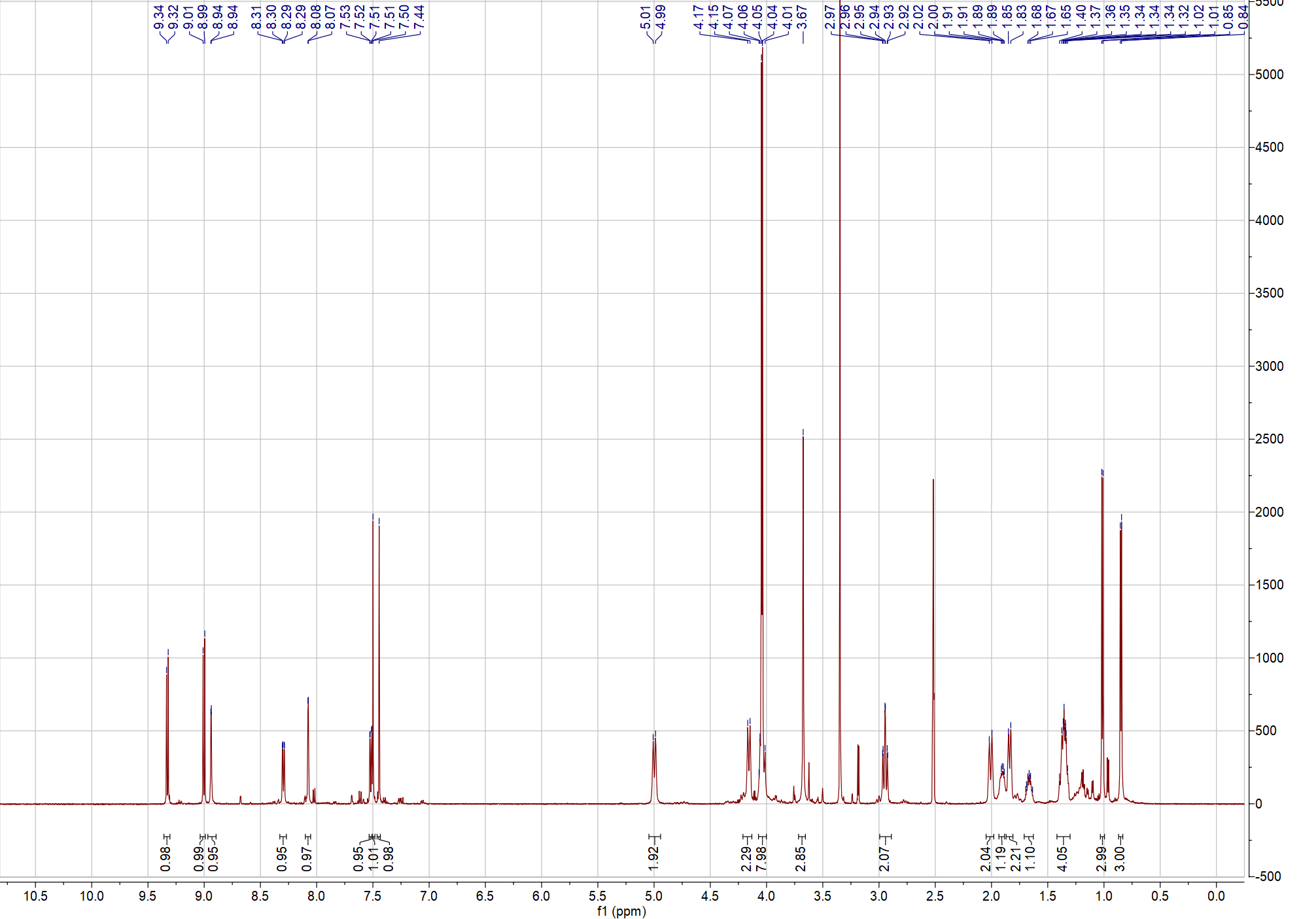


**Fig. S17.** ^1^H NMR spectrum of **M3**

**
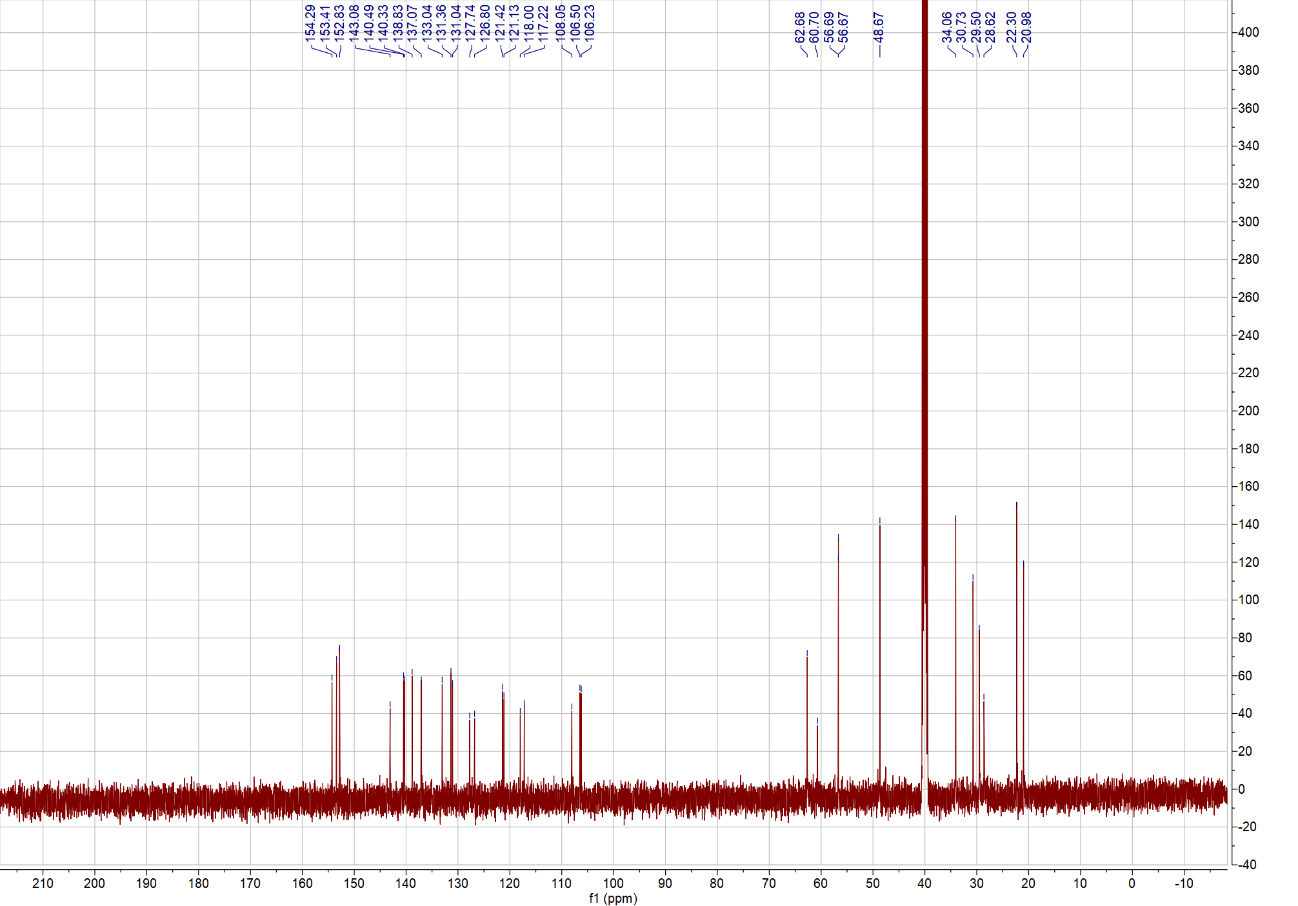
**

**Fig. S18.** ^13^C NMR spectrum of **M3**

**Fig. S19.** HRMS spectrum of **M3**

**
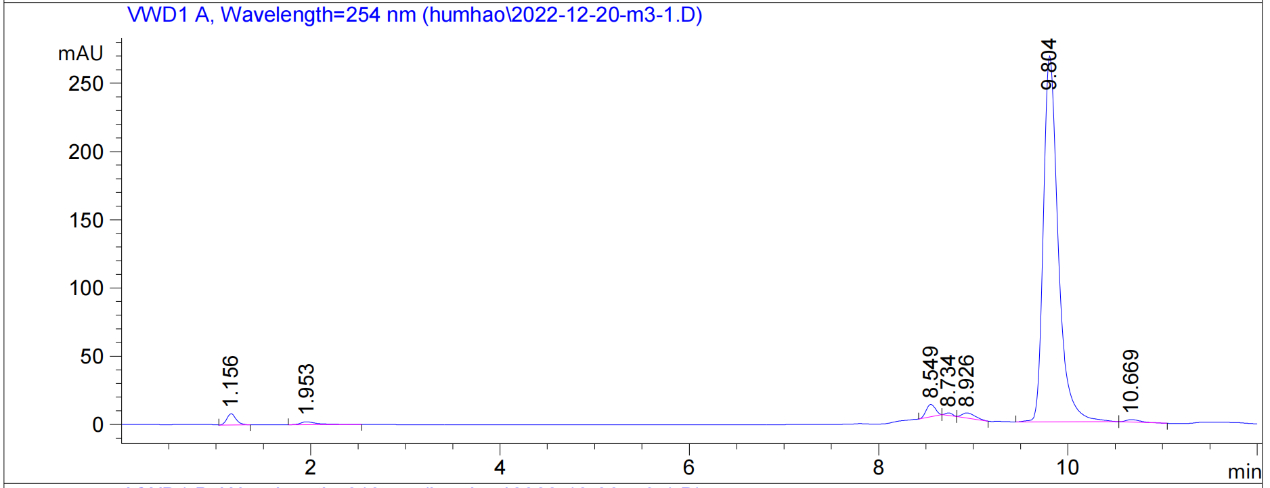
**

**Fig. S20.** HPLC spectrum of **M3**


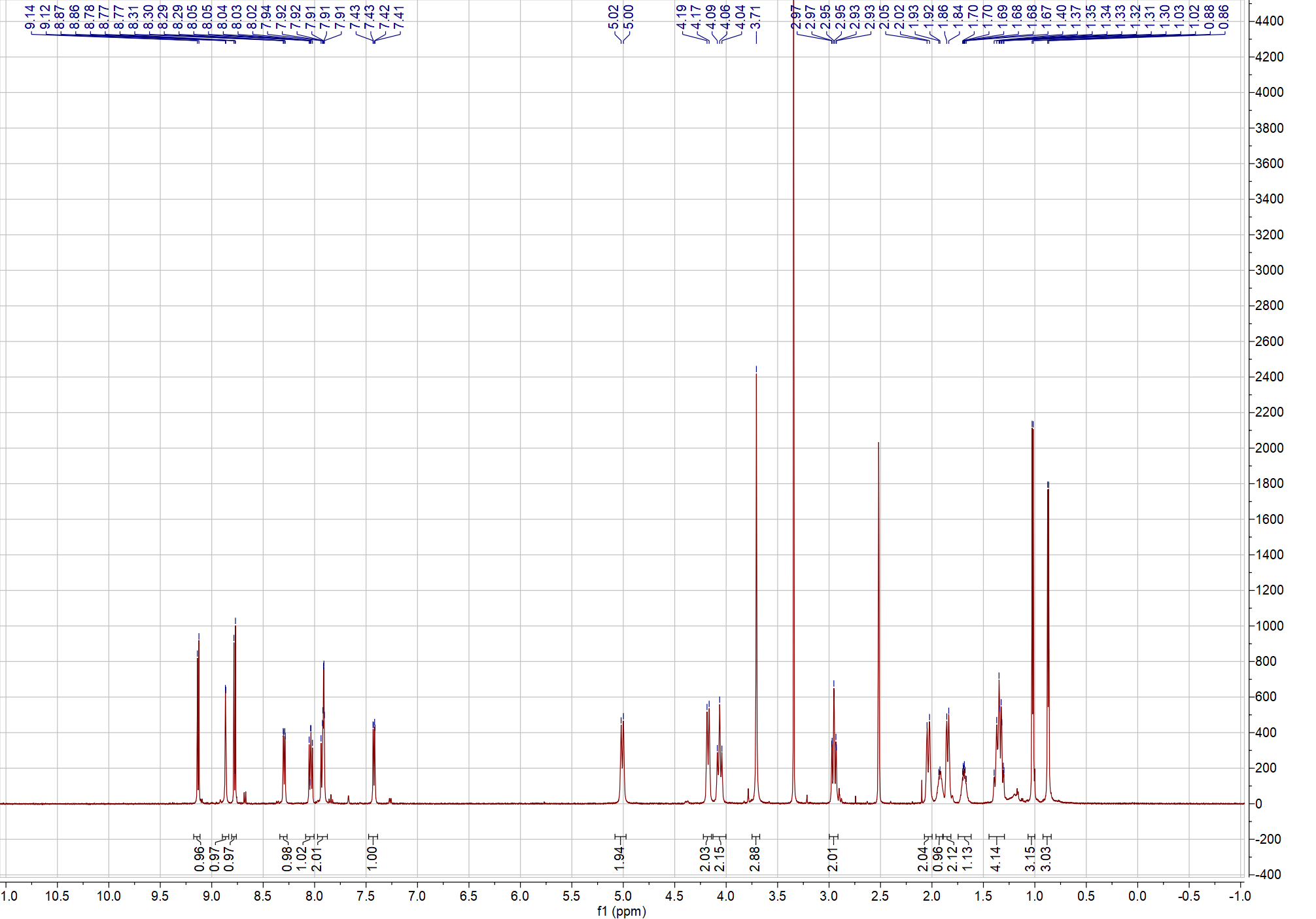


**Fig. S21.** ^1^H NMR spectrum of **M4**

**
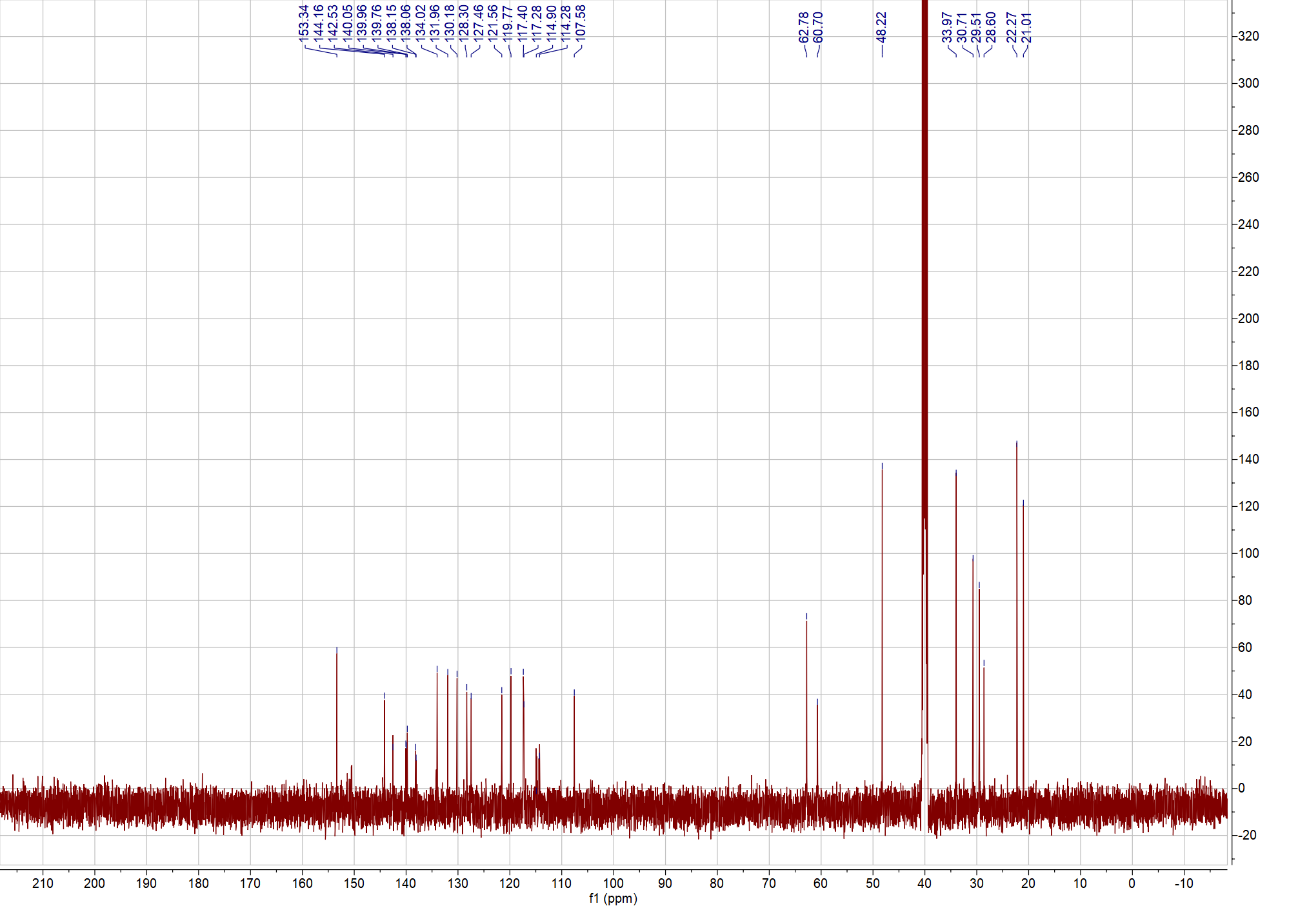
**

**Fig. S22.** ^13^C NMR spectrum of **M4**

**Fig. S23.** HRMS spectrum of **M4**

**
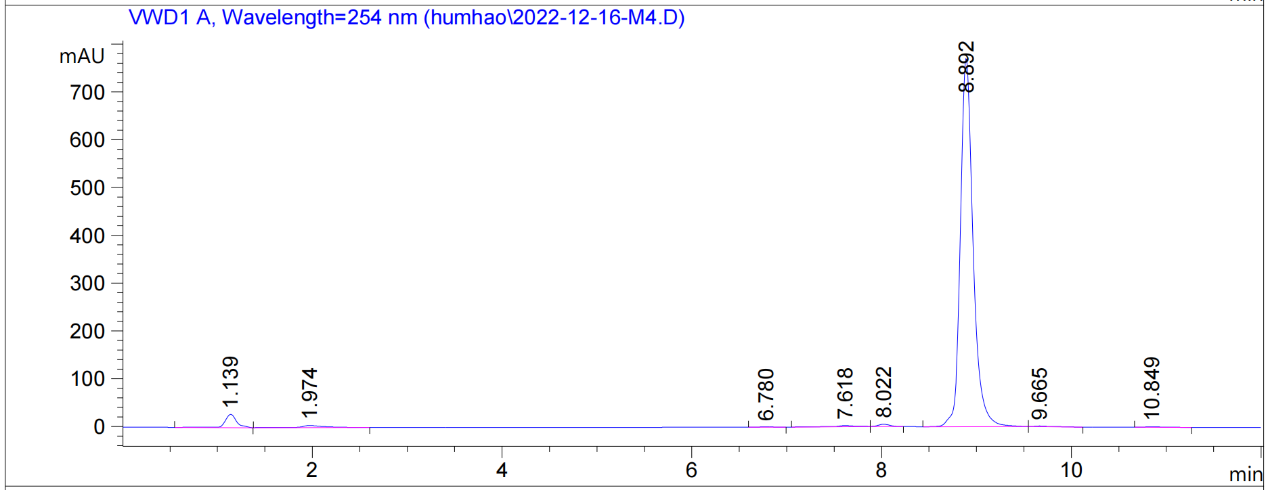
**

**Fig. S24.** HPLC spectrum of **M4**


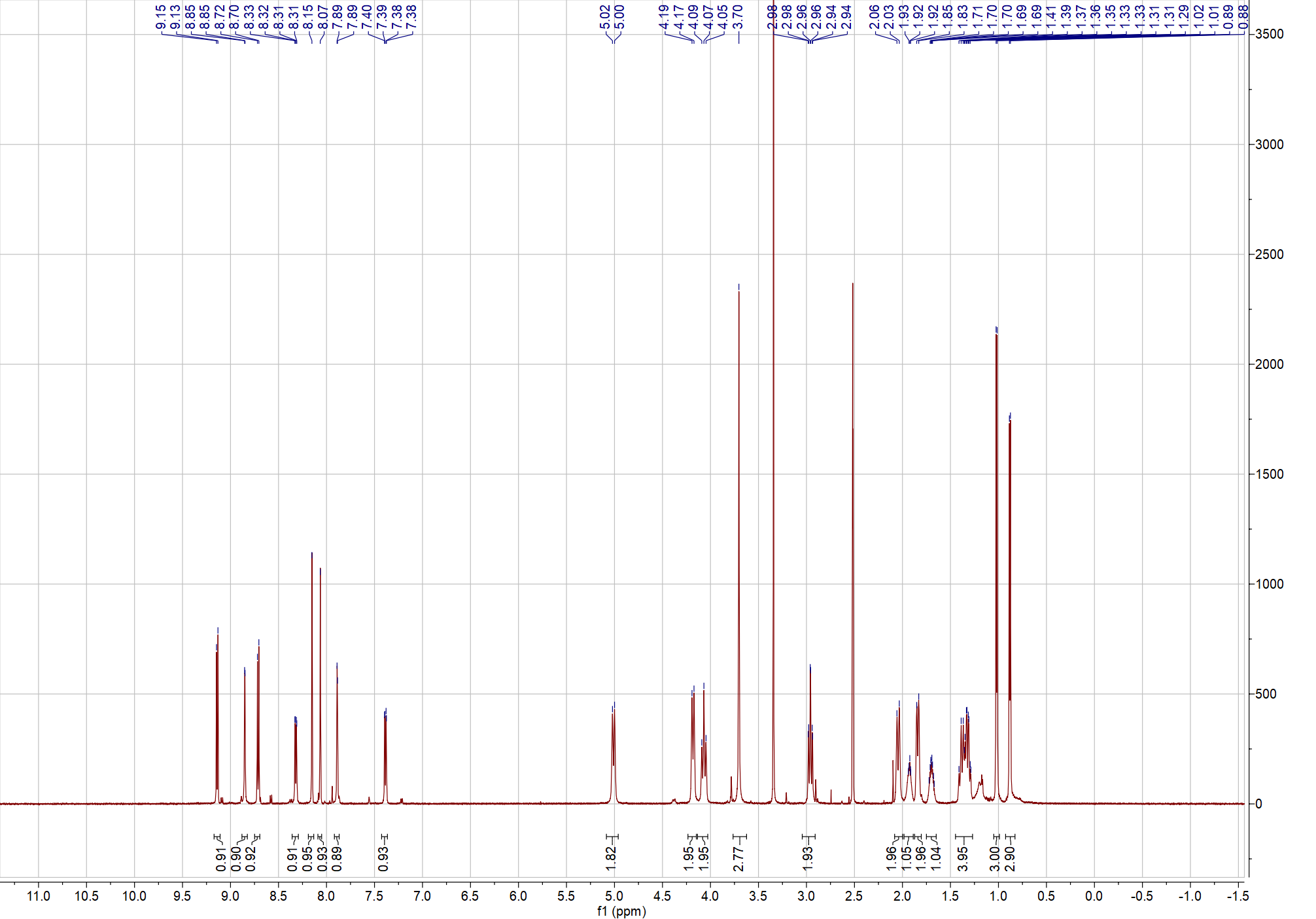


**Fig. S25.** ^1^H NMR spectrum of **M5**

**
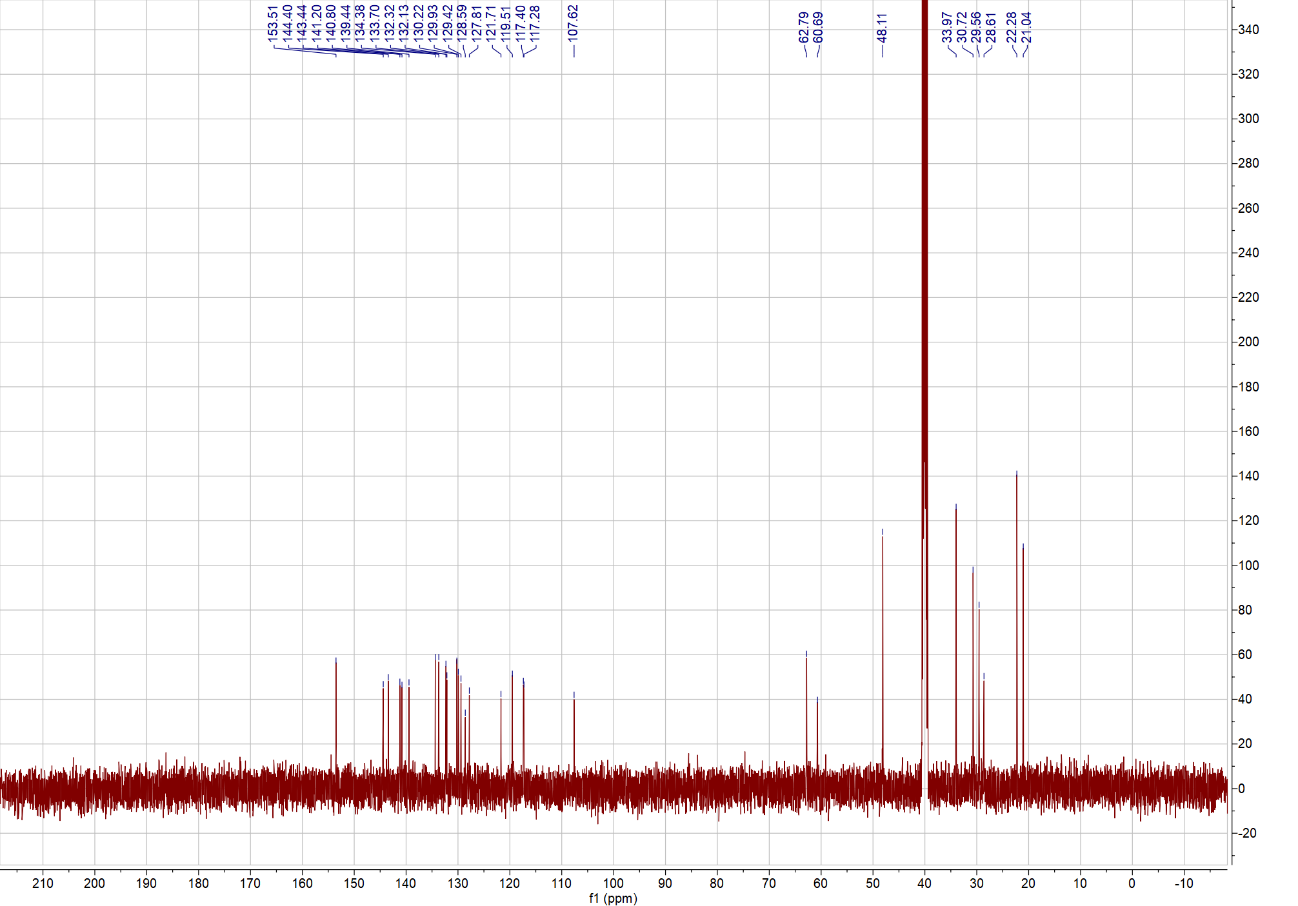
**

**Fig. S26.** ^13^C NMR spectrum of **M5**

**Fig. S27.** HRMS spectrum of **M5**

**
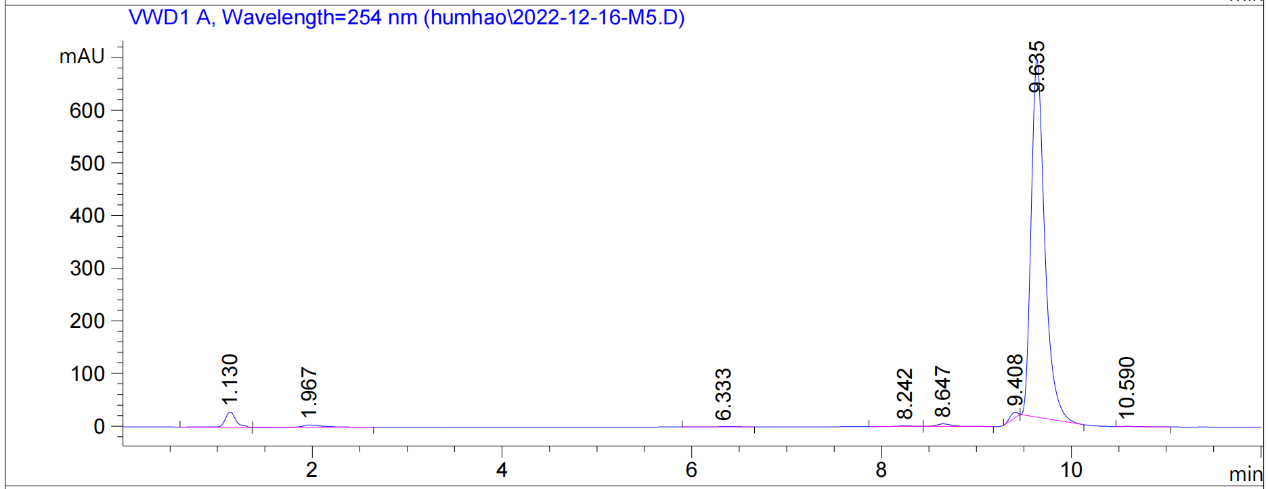
**

**Fig. S28.** HPLC spectrum of **M5**
